# Circular RNA MTCL1 promotes advanced laryngeal squamous cell carcinoma progression by inhibiting C1QBP ubiquitin degradation and mediating beta-catenin activation

**DOI:** 10.1186/s12943-022-01570-4

**Published:** 2022-04-02

**Authors:** Zheng Wang, Anqi Sun, Aihui Yan, Jian Yao, Haibo Huang, Ziming Gao, Tao Han, Jia Gu, Ni Li, Huizhe Wu, Kai Li

**Affiliations:** 1 Department of Otorhinolaryngology, the First Affiliated Hospital of China Medical University, Shenyang, 110001 China; 2 Department of Surgical Oncology and General Surgery, Key Laboratory of Precision Diagnosis and Treatment of Gastrointestinal Tumors, Ministry of Education, the First Affiliated Hospital of China Medical University, Shenyang, 110001 China; 3 Division of Molecular Signaling, Department of the Advanced Biomedical Research, Interdisciplinary Graduate School of Medicine, University of Yamanashi, Chuo, Yamanashi, Japan; 4 Department of Oncology, the First Affiliated Hospital of China Medical University, Shenyang, 110001 China; 5 Office of Cancer Screening, National Cancer Center, National Clinical Research Center for Cancer, Cancer Hospital, Chinese Academy of Medical Sciences Key Laboratory for National Cancer Big Data Analysis and Implement, Chinese Academy of Medical Sciences and Peking Union Medical College, Beijing, 100021 China; 6 Department of Pharmacology, School of Pharmacy, China Medical University, Shenyang, 110122 China

**Keywords:** circRNA MTCL1, C1QBP, Ubiquitylation, *β*-Catenin, Laryngeal neoplasms

## Abstract

**Background:**

Circular RNAs (circRNAs) are involved in regulatory processes of ubiquitination and deubiquitination in various tumors at post-transcriptional epigenetic modification level. However, the underlying mechanism and its biological functions of circRNAs in the advanced laryngeal squamous cell carcinoma (LSCC) remain obscure.

**Methods:**

RNA sequencing and quantitative real-time PCR (qRT-PCR) assays were applied to screen for circRNAs differentially expressed in LSCC tissues and cell lines. The candidate RNA-binding proteins and target signalling pathway were detected by RNA pull-down and mass spectrometry, in situ hybridization (ISH), immunohistochemistry (IHC), qRT-PCR assays, and bioinformatics analysis. The functional roles of these molecules were investigated using in vitro and in vivo experiments including EdU, transwell, wound healing, western blot assays, and the xenograft mice models. The molecular mechanisms were identified using RNA pull-down assays, RNA immunoprecipitation (RIP), Co-IP, ISH, Ubiquitination assay, bioinformatics analysis, and the rescue experiments.

**Results:**

Here, we unveil that microtubule cross-linking factor 1 circRNA (circMTCL1, circ0000825) exerts its critical oncogenic functions by promoting complement C1q-binding protein (C1QBP)-dependent ubiquitin degradation and subsequently activating Wnt/*β*-catenin signalling in laryngeal carcinoma initiation and development. Specifically, circMTCL1 was remarkably up-regulated in the paired tissues of patients with LSCC (*n* = 67), which predicted a worse clinical outcome. Functionally, circMTCL1 exerted oncogenic biological charactersistics by promoting cell proliferative capability and invasive and migrative abilities. Ectopic circMTCL1 augumented cell proliferation, migration, and invasion of LSCC cells, and this effect could be reversed by C1QBP knocking down in vitro and in vivo. Mechanistically, circMTCL1 directly recruited C1QBP protein by harboring the specific recognized sequence (+ 159 − + 210), thereby accelerating the translation of C1QBP expression by inhibiting its ubiquitin–proteasome-mediated degradation. Importantly, the direct interaction of C1QBP with *β*-catenin protein was enhanced via suppressing the *β*-catenin phosphorylation and accelerating its accumulation in cytoplasm and nucleus.

**Conclusion:**

Our findings manifested a novel circMTCL1-C1QBP-*β*-catenin signaling axis involving in LSCC tumorigenesis and progression, which shed new light on circRNAs-ubiquitous acidic glycoprotein mediated ubiquitin degradation and provided strategies and targets in the therapeutic intervention of LSCC.

**Supplementary Information:**

The online version contains supplementary material available at 10.1186/s12943-022-01570-4.

## Introduction

Laryngeal squamous cell carcinoma (LSCC) remains one of the most common malignancies of head and neck accounting for approximately 20% of all cases [1]. Up to 60% of LSCC patients presents with advanced clinical stage (III or IV) at diagnosis [2]. Despite significant advancements have been made on the treatment strategies including surgery, chemotherapy, radiotherapy, and immunotherapy, five-year survival rate for LSCC has decreased over the past decades [3]. Thus, it is urgent to excavate the novel reliable targets for early diagnosis and therapeutic biomarkers of prognostic assessment for LSCC patients. Recent accumulating evidences supported that non-coding RNAs (ncRNAs) such as circular RNAs (circRNAs), long non-coding RNAs (lncRNAs), and microRNAs (miRNAs) became the hallmark of tumirgenesis, which acted as master gene modulators contributing to the initiation and development of various tumors at the transcriptional and post-transcriptional epigenetic levels [4–7]. However, the underlying molecular mechanisms that control tumorigesis and progression remain unclear, particularly in LSCC [8, 9].

CircRNAs are usually produced by non-canonical back-splicing events characterized with covalently closed structure lacking of 5’caps and 3’poly(A) tails [10, 11]. Although the first circRNA molecule has been identified in 1976, these molecules were initially regarded as aberrant byproducts and no cellular functions have been identified until recently [12, 13]. Up to date, mounting evidences demonstrated that circRNAs exert their critical biological roles as protein sponges or decoys, protein scaffolding to regulate the targets expression in the context of tumor pathologenesis and progression [14–16]. Exemplified by circ-SHPRH, a protein templates driven by internal ribosome entry site (IRES) elements acts as the SHPRH protein decoy to prevent DTL-mediated ubiquitination degradation, thereby inhibiting glioma development [17]. Meanwhile, circ-Foxo3 functions as protein scaffolding binding to specific proteins such as CDK2, p21, MDM2, and p53 to arrest the expresson or induce the degradation of these indicators [18]. Thus, circRNAs showed vital regulatory effects in the occurrence and progression of a variety of tumors by affecting cell cycle, apoptosis, invasion, and metastasis, etc. [19–21]. However, little is known about the biological functions, and regulatory mechanisms of circRNAs in LSCC.

As one of the RNA-binding proteins, C1QBP is a ubiquitous and multifunctional protein, which interacts with RNA, subsequently exerting the regulatory functions [22]. C1QBP expression is dysregulated in several tumours and is involved in promoting cell migration and tumour growth in melanoma [23]. In cervical squamous cell carcinoma, C1QBP was found dramatically down-regulated and inhibited the cell viability, migration and proliferation [24]. Alternatively, C1QBP was also found to regulate cell proliferation, invasion, migration and apoptosis by activating the phosphoinositide 3-kinase (PI3K)/mitogen-activated protein kinase (MAPK) pathway in pancreatic cancer [25]. It must be noted that concrete examples of regulation pattern of circRNAs with C1QBP in response to stimuli, especially in the context of ubiquitin–proteasome-mediated degradation, have not been reported yet. Therefore, this prompt us to explore the functional regulation manner and molecular mechanisim of circRNAs interacting with C1QBP in LSCC tumorigenesis and development.

In our present study, a novel upregulated circRNA (circular RNA MTCL1, chr18:8718421–8,720,494) circMTCL1 was identified in advanced LSCC tissues compared with matched adjacent normal mucosa (ANM) tissues via high-throughput sequencing analysis. High circMTCL1 level showed significantly relationship with poor clinical outcomes for LSCC patients. Notably, oncogenic circMTCL1 directly bond to ubiquitous acidic glycoprotein C1QBP, promoted its recruitment and inhibited ubiquitin degradation of C1QBP and subsequently accelerating active *β*-catenin and its associated signalling pathway, consequently exerted an oncogene function in LSCC cells. Thus, our results present a previously unappreciated importance of post-translational dysregulation, as mediated by circMTCL1 in a ubiquitin-proteasome-dependent manner, which provides new approach to understand how circRNAs and the corresponding RNA-binding proteins regulated the targets and supplys LSCC prevention and treatment with novel targets and directions.

## Methods

### Patients and specimens

A total of 67 advanced LSCC tissue samples were collected with matched paracancerous control tissues from patients with LSCC treated at our hospital in 2016–2017. All patients provided written informed consent before enrolment, and this study was approved by the ethics committee of our university following with tenets of the Declaration of Helsinki (Ethical code, AF-SOP-07-1.1-01). LSCC was diagnosed through histological examinations conducted by three experienced pathologists. Clinicopathological classification and staging were performed following the American Joint Committee on the cancer classification criteria.

### Cell culture

LCC and LLN LSCC cell lines were obtained from the Institute of Basic Medical Science, Chinese Academy of Medical Sciences & School of Basic Medicine Peking Union Medical College. Then, the LSCC cell line Tu212 and normal cell line HaCaT were obtained from the Laryngeal Cancer Research Office (First Affiliated Hospital of China Medical University, Shenyang, China). All of these cell lines were cultured in Roswell Park Memorial Institute (RPMI) using a 1640 medium (Gibco,New York, USA) containing 10% foetal bovine serum (FBS) (Bovogen, Melbourne, Australia) and were incubated in a humidified atmosphere containing 5% CO_2_ at 37 °C. All cell lines were verified by short tandem repeat profiling using routine mycoplasma testing within 6 months of receipt.

### Mouse model

All animal experimental procedures were conducted based on the guidelines of the National Institutes of Health for the Care and Use of Laboratory Animals and were approved by the Animal Care and Use Committee of our university (Ethical code, KT2019030). The stably transfected TU212 cells (2 × 10^6^ cancer cells/mouse, five/group) were used for in vivo tumour growth experiments and TU212 cells (0.5 × 10^6^ cancer cells/mouse, five/group) for experimental metastatic studies were subcutaneously injected into the back or caudal veins of 4-week-old male BALB/c mice. Tumour volume assessments and in vivo imaging were performed weekly. The mice were sacrificed after 4 or 8 weeks, and tumour tissues were excised and used for pathological examinations and immunofluorescent labelling experiments.

### RT-PCR and real-time quantitative RT-PCR

A TRIzolkit (15,596,018, Thermo Fisher, Waltham, USA) was used to extract the total RNA. To detect the circRNA expression, the RNase R (3 U/mg, Epicenter,Madison, WI) digestion was performed at 37 °C for 20 min. Reverse transcription was performed using a high-capacity cDNA reverse transcription kit (4,374,967, Thermo Fisher, Waltham, MA, USA). TB Green Premix Ex Taq II (RR820A, TaKaRa, Tokyo, Japan) and primers were used for RT-PCR based on the manufacturer’s instructions. Transcript levels were analysed using the2^-△△Ct^ method. The primers used in this study are shown in Table S10.

### RNase R treatment

To identify circular characteristics and assess the circMTCL1 stability, the total RNA (2 μg) was incubated with or without 3 U/μg RNase R (6 U) (Epicentre, San Diego, CA, USA) at 37 °C for 30 min. Random primers (RR037A, Takara, Tokyo, Japan) and oligodT primers (RR037A, Takara, Tokyo, Japan) were used to reverse transcribe circMTCL1 and glyceraldehyde 3-phosphate dehydrogenase (GAPDH), respectively. Specific divergent (Sangon, Shanghai, China) and convergent primers (Sangon, Shanghai, China) were used for PCR amplification. PCR products were quantified using 2% agarose gel electrophoresis (R0491, Thermo Fisher, Waltham, MA, USA). All primer sequences used in this study are listed in Table S10.

### Western blotting

The radioimmunoprecipitation buffer (R0030, Solarbio, Beijing, China) on ice was used to lyse for 30 min. An ultraviolet spectrophotometer (912A0888, Thermo Fisher Scientific, Waltham, MA, USA) was used to quantify protein concentrations. Lysates were resolved using the SDS-PAGE and transferred onto the polyvinylidene fluoride membranes (Millipore, Bedford, MA, USA). The membranes were blocked with a 5% FBS sealant for 1 h at room temperature and incubated with high-affinity primary antibodies against C1QBP (ab232712, 1:1000, Abcam, Cambridge, MA, USA), β-catenin (ab6302, 1:1000, Abcam, Cambridge, MA, USA), phosphorylated β-catenin (ab75777, 1:500, Abcam, Cambridge, MA, USA), GSK3β (ab93926, 1:2000, Abcam, Cambridge, MA, USA), phosphorylated GSK3β (ab75814, 1:2000, Abcam, Cambridge, MA, USA), ZNF24 (ab176589, 1:2000, Abcam, Cambridge, MA, USA), UBAP2L (ab70319, 1:2000, Abcam, Cambridge, MA, USA), CBX8 (ab182627, 1:2000, Abcam, Cambridge, MA, USA), MARCKSL1 (ab163921, 1:2000, Abcam, Cambridge, MA, USA), CDKN2AIP (ab140519, 1:2000, Abcam, Cambridge, MA, USA), TOX3 (ab216614, 1:2000, Abcam, Cambridge, MA, USA), ATP5B (ab14730, 1:2000, Abcam, Cambridge, MA, USA), APC (2504, 1:1000, CST, Danvers, MA, USA), CK1 (2655, 1:1000, CST, Danvers, MA, USA) and GAPDH (ab181602, 1:2000, Abcam, Cambridge, MA, USA) overnight at 4 °C, followed by incubation with a horseradish peroxidase (HRP)-conjugated secondary monoclonal antibody (ab67281:2000, Abcam, Cambridge, MA, USA) for 1 h at room temperature on the next day. Then, a chemiluminescence system (1,708,280, Bio-Rad, Hercules, California, USA) was used to detect proteins.

### Ubiquitination assay

The cell-permeable proteasome inhibitor MG132 (20 μM; C2211, sigma-adlrich, USA) was added to cellcultures for 6 h, followed by an immunoprecipitation lysis buffer containing protease and phosphatase inhibitors for 30 min. The lysates were immunoprecipitated with an anti-C1QBP antibody (ab101267, 1:500, Abcam, Cambridge, MA, USA) or immunoglobulin G (IgG) (ab6709, 1:500, Abcam, Cambridge, MA, USA) overnight at 4 °C on a rotating pattern. An antibody against ubiquitin (10201–2-AP, 1:1000 Proteintech, Rosemount, Minnesota, USA) was used to measure C1QBP ubiquitination. The immunoprecipitated proteins were quantified usingWB.

### RNA sequencing and analysis

The total RNA was extracted using the TRIzol reagent kit (15,596,018, Thermo Fisher, Waltham, USA). The RNA quality was assessed using an Agilent 2100 Bioanalyzer instrument (Paloalto, California, USA). An Agilent 2100 Bioanalyzer and an ABI StepOnePlus RT-PCR System (Thermo Fisher, Waltham, USA) were used to quantify the libraries. Transcriptome sequencing was performed using an Illumina HiSeq X Ten platform from Beijing Genomics Institute (Shenzhen, China) on 4 of 67 paired LSCC and adjacent normal tissues. Differentially expressed transcripts were identified using the following criteria: |log2 (fold change)| > 1 and *P*-value < 0.05. The CIRC explorer (*http://yanglab.github.io/CIRCexplorer/*) and circBase (*http://www.circbase.org*/) databases were used to predict and obtain circRNA sequences.

### RNA pulldown and mass spectrometry

A random circMTCL1 probe was labelled using a Pierce RNA 3′ End Desthiobiotinylation Kit (20,163; Thermo Fisher, Waltham, USA); the mixture contained T7 RNA polymerase, and therefore, streptomycin affinity beads (50 μL) were added to the reaction buffer. First, the labelled RNA was diluted in a solution containing Tris (20 mM, pH 7.5); then, the cell lysates containing 10 million cancer cells were added at 4 °C for 50 min. The WB and mass spectrometry (QEXACTIVE, Thermo Fisher, Waltham, USA) were used to identify the retrieved proteins.

TU212 cells with or without circMTCL1 knockdown seeded in a 10-cm dish at 70–80% confluency were harvested by trypsinisation. Cytoplasmic extraction was isolated by 500 μl 1 × hypotonic buffer and 10% NP-40. About 40 μM of circMTCL1-WT or circMTCL1-Mut biotin-labelled and circMTCL1-antisense probes (negative control) were conjugated to streptavid in agarose resin beads (Thermo Fisher Scientific) through a 4-h incubation at 4 °C, respectively, and then washed thrice. Furthermore, it was incubated with pre-cleared nuclear extraction in RIP buffer (150-mM KCl, 25-mM Tris (pH 7.4), 5-mM EDTA, 0.5-mM DTT, 0.5% NP40 and 1× protease inhibitor) at 4 °C overnight. The protein was isolated using 40 μl 1 × SDS protein lysis at 95 °C for 10 min and 13,000 g centrifuged for 10 min. Co-immunoprecipitated and input proteins were analysed through SDS-PAGE separation. RNA probes used for RNA pull-down assays are listed in Supplementary file 1 (Table S14).

### RNA fluorescence in situ hybridisation (RNA-FISH)

The cell slides were fixed with 4% paraformaldehyde (PFA) for 20 min and added with proteinase K (20 μg/mL) for digestion. Then, hybridisation was performed using the hsa-circ-0000825 probe (sequence: 5′-DIG-GTTCATCTAACTCATCCTCTTTCAGTCTCTCC-3′) at a 6-ng/μL concentration overnight. Slides were incubated with an anti-digoxigenin (DIG)-HRP antibody, and the nuclei were counterstained with 4′,6-diamidino-2-phenylindole (DAPI). Images were collected using a Nikon inverted fluorescence microscope (Ti2-U, Tokyo, Japan).

### Subcellular RNA fractionation

The nuclear and cytosolic fractions were separated following the manufacturer’s instructions using a PARIS KIT (AM1921, Invitrogen, CA, USA). The circMTCL1 expression was analysed using RT-qPCR, and GAPDH with U6 was used as a fractionation indicator.

### Transfections and vector constructions

For circMTCL1 or C1QBP overexpression in vitro, 436 or 858 bp cDNA fragments containing the circMTCL1 or C1QBP were cloned into a pLCDHor Y0009367 vector (GenePharma, Suzhou, China) between EcoRI and BamHI or between BspEI-BglII restriction sites. For circMTCL1 or C1QBP knockdown, cells were transfected with siRNAs (GenePharma, Suzhou, China) against circMTCL1 or C1QBP. siRNA sequences are shown in Tables S8 and S9.The plasmids or siRNAs were transiently transfected into cells following the manufacturer’s instructions using an X-treme GENE siRNA (4,476,093,001, Roche, Basel, Switzerland) or DNA Transfection Reagent (6,366,244,001,Roche, Basel, Switzerland).

For in vivo studies, a lentiviral vector containing circMTCL1 shRNA was synthesised in Hanbio (Shanghai, China), which was used for TU212 cell transfection to establish stable cell lines overexpressing circMTCL1. Lentiviral transfections were performed following the manufacturer’s instructions.

### EdU assays

EdU assays were performed to evaluate the cells’ proliferation ability based on the manufacturer’s instructions regarding the use of the Cell Light EdU Apollo488 In Vitro Imaging Kit (C10338–3, RiboBio, Guangzhou, China). Briefly, transfected cells were seeded into 12-well plates at a density of 1 × 10^4^ cells/well. After adding EdU, the cells were cultured for 2 h, fixed with 4% PFA and stained with the kit following the manufacturer’s instructions. Images were captured using an Olympus microscope (IX53, Olympus, Tokyo, Japan).

### Cell migration and invasion assay

The migratory and invasive abilities of the cells were measured in 24-well Transwell chambers with 8-μm pores (Corning, NY, USA). For migration assays, transfected cells with a density of 5 × 10^4^ cells/200 μL were added to the upper chamber, whereas the RPMI medium supplemented with 10% FBS was added to the lower chamber. After a 24-h incubation, cells in the lower chamber were fixed with 4% PFA and stained with 0.1% crystal violet (Weijia Biology Science and Technology Co., Guangzhou, China). For invasion assays, a polycarbonate membrane pre-coated with Matrigel matrix (354,248, BD Bioscience, Franklin Lakes, New Jersey, USA) was used. For quantification, a microscope (IX51, Olympus, Japan) was used to count cells in five randomly selected fields. All experiments were independently performed thrice. Data are shown as mean ± standard deviation (SD).

### Wound healing assay

Transfected cells were seeded in 6-well plates. The plates were scratched using a 200-μL pipette tip when the cells reached confluency, with at least three lines crossing each well, followed by incubation with 1% FBS. Wound healing was imaged at 0 h and 48 h using a microscope (IX51, Olympus, Japan) and was analysed using Image J 1.8.0.

### Crosslinking RIP assay

The RIP assay for C1QBP was performed at room temperature to quantify the protein expression using the Magna RIP RNA-Binding Protein Immunoprecipitation Kit (17–701, Millipore, Billerica MA, USA) following the manufacturer’s instructions. TU212 cells were incubated using magnetic beads conjugated with C1QBP-specific antibody (ab24733, Abcam, Cambridge, MA, USA) or control IgG (ab6789, Abcam, Cambridge, MA, USA). The immunoprecipitated RNA was reverse-transcribed into the cDNA and was amplified using qPCR to identify any circMTCL1. The primers are listed in Table S10.

### Co-IP assays

Co-IP assays for C1QBP and β-catenin were performed at room temperature. Lysates containing ten million cells were incubated with 1-μganti-C1QBP antibody (ab24733, Abcam, Abcam, Cambridge, MA, USA) and anti-β-catenin antibody (ab32572, Abcam, Abcam, Cambridge, MA, USA) at 4 °C overnight with slow rotation. The antigen-antibody complexes were retrieved using protein A beads (8687S, CST, Danvers, MA, USA) and then washed three times with pre-cooled phosphate-buffered saline. IP complexes were eluted with Laemmli buffer and subjected to WB.

### IHC

All tissues were fixed with 10% neutral formaldehyde solution before serial sectioning at 2 μm using a microtome (RM2016, leicaSOLMS, Germany). Pathological sections were deparaffinised with xylene and graded ethanol. Subsequently, tissues were incubated using a rabbit monoclonal C1QBP antibody (1:200, Abcam, Cambridge, MA, USA) at 4 °C, followed by IgG incubation (ab150113, Abcam, Abcam, Cambridge, MA, USA) following the manufacturer’s instructions for 2 h at room temperature. A scoring system was used to assess the degree of expression based on the percentage of positively labelled cells (0, 0%; 1, < 10%; 2, 10–50% and 3, > 50%) and staining intensity (0, no staining; 1, weak staining; 2, moderate staining and 3, strong staining).

### Statistical analysis

A two-tailed Student’s *t*-test was used to determine statistically significant between-group differences. Survival rates were calculated using the Kaplan–Meier method and analysed by the log-rank test. Chi-squared test was performed to identify associations between circMTCL1 expression and clinicopathological parameters in our patients with LSCC. Data are presented as mean ± SD. Statistical analyses were performed using the SPSS 19.0 software (version 22.0; Chicago, IL, USA) and GraphPad Prism 7 software (GraphPad, USA). A*P* < 0.05 indicates statistical significance.

## Results

### Expression, subcellular localisation and prognostic value of circMTCL1 in the LSCC cells and tissues

**Fig. 1:**
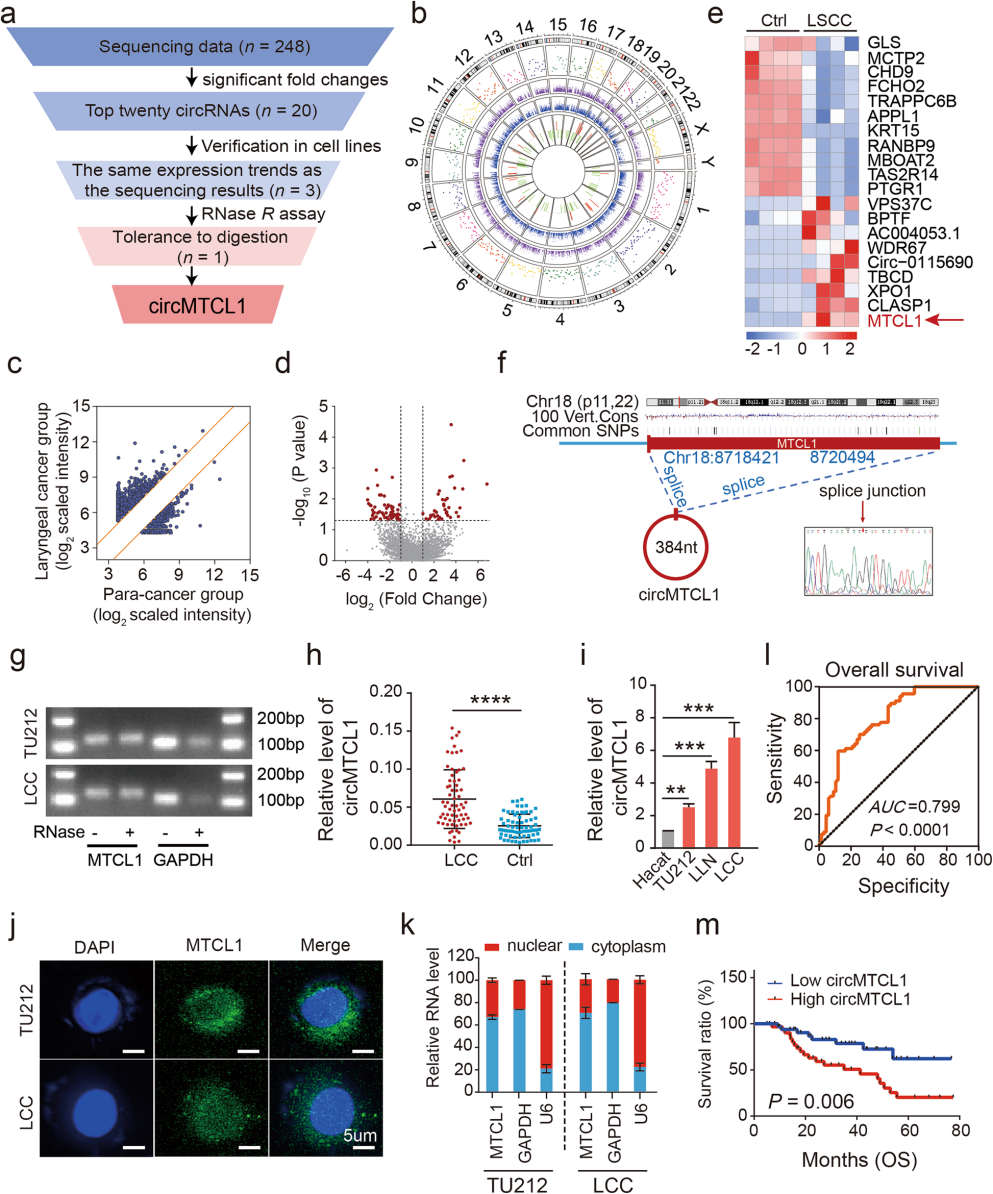
The characteristics of circMTCL1 in LSCC cells and tissues. **a**, The flowchart illustrating the selecting processes of circMTCL1 based on the sequencing data. **b**, A circular diagrams from the most inner circle to the most outer circle represent the log2 fold change value of up-regulated or down-regulated differentially expressed circRNAs of the LSCC compared with matched adjacent-tumor control tissues (*n* = 4, *P* < 0.05), the gene expression value of matched adjacent-tumor control tissues, the gene expression value of LSCC, the different chromosome location of the genes in different colors, and the ruler of chromosome. **c**, The scatter plots of circRNAs expression among included 4 pairs of LSCC tissues and their matched tumour-adjacent normal samples. The dashed lines represent 2.0-fold change. **d**, The volcanic plot of circRNAs expression among included 4 pairs of LSCC tissues and adjacent-tumor control tissues (*n* = 4, Log 2 FC = 2.0, *P* < 0.05). **e**, The hierarchical clustering heat map showing the most differentially expressed circRNAs in LSCC and corresponding paired adjacent-tumor control tissues, selected top 10 up-regulated or down-regulated genes (*n* = 4, *P* < 0.05). Red in heat map represents upregulation. Blue represents downregulation. **f**, The ideograph illustrating the genomic location and splicing mode of circMTCL1. The splicing junction site was confirmed by sanger sequencing. **g**, The RNA enzyme digestion test was performed to validate the stability of circMTCL1 in TU212 and LCC cells. **h**, The expression level of circMTCL1 in 67 pairs of LSCC tissues and their ANM samples was assessed by qRT-PCR assays. Values are the mean ± s.d. of *n* = 3 independent experiments. **i**, The expression level of circMTCL1 in normal control HaCaT cell and LSCC cells including TU212, LCC, LLN was detected by qRT-PCR assays. Values are the mean ± s.d. of *n* = 3 independent experiments. **j**, Localization of circMTCL1 detected by FISH assay in TU212 and LCC cells. Scale bar = 50 μm. **k**, The expression level of circMTCL1 within the TU212 and LCC cells amplifying with isolated RNA from the cytoplasmic contents and cell nuclei was detected by qRT-PCR assays. Values are the mean ± s.d. of *n* = 3 independent experiments. **l**, The receiver operating characteristic (ROC) curve showing the diagnostic cut-off value of circMTCL1 expression. **m**, The log-rank test was explored to investigate the impact of the circMTCL1 expression level on the survival of patients with LSCC. *, *P* < 0.05; **, *P* < 0.01; ***, *P* < 0.001; ****, *P* < 0.0001

To explore the dysregulated circRNAs, differential expression profiling analyses were performed between LSCC patient samples and their matched ANM tissues (*n* = 4) based on RNA sequencing (Fig. 1a and Table S1). The significant differences between these two groups were set at a ≥ log2-fold change and *P*-value of < 0.05. Primarily, the expression differences profiling was widely distributed across all chromosomes, including the sex chromosomes (X and Y) (Fig. 1b). In total, 6856 circRNA transcripts were identified from the sequencing data. Of these, 3195 and 3661 were up- and down-regulated, respectively, in this cohort (Fig. S1a). Specifically, 248 of circRNA transcripts were differentially expressed, and 158 and 90 of them were up- and down-regulated, respectively (Fig. 1c, d and Fig. S1a). Among these, 20 circRNAs exhibiting the highest up- or down-regulated trends were selected for further investigations with nine circRNAs having more than two read counts (Table S2 and Fig. S1b). Further validation using quantitative reverse transcription-polymerase chain reaction (qRT-PCR) assays confirmed that three circRNAs in LSCC cell lines demonstrated consistent trends in expression levels as those of the sequencing data (Table S3 and Fig. S1c). Two circRNAs, circMTCL1 and adaptor protein phosphotyrosine interacting with the pH domain and leucine zipper 1 (APPL1) circRNA (circAPPL1), exhibited similar expression trends in sequenced tissues as that of sequencing data (Table S4 and Fig. S1d). Ultimately, circMTCL1 was found to be resistant to ribonuclease *R* (RNase *R*) digestion (Fig. 1a, g and Fig. S1e), and circMTCL1 was selected for further examinations.

Thereafter, the heatmap clustering analysis containing the top 20 dysregulated circRNAs expression profiles revealed that circMTCL1 was significantly up-regulated, whereas log_2_ FC value was 1.48 (Fig. 1e and Table S5). The circMTCL1 sequence was obtained from the circRNA database circBase (*www.circbase.org*), comprising 384 nucleotides (nt), and was spliced from the *MTCL1* gene, rooted in chr18:8718421–8,720,494(Fig. 1f). Furthermore, sanger sequencing confirmed the existence of circMTCL1 using divergent primers in TU212 cells (Fig. 1f). The base sequence across the junction site was amplified, and the results were confirmed based on the circBase data (Fig. 1f)**.**

To further validate the differences in circMTCL1 expression between the tissues of patients with LSCC and their matched tumour-adjacent normal samples, the circMTCL1 expression was assessed in 67 pairs of LSCC tissues and their ANM samples by qPCR assays. As expected, the circMTCL1 expression was significantly up-regulated in the LSCC tissues (Fig. 1h). Similar results were confirmed in three LSCC cell lines including TU212, laryngeal cell carcinoma (LCC) and laryngeal lymph node (LLN) cell lines as compared with the immortalised human keratinocyte (HaCaT) normal control cells (Fig. 1i). Thus, these findings manifested that circMTCL1 was significantly up-regulated in LSCC cells and tissues. Furthermore, the expression level of circMTCL1 was validated in the other populations in the GEO database. Similar results showed that circMTCL1 was also upregulated in the most of the other cancers, including gastric cancer, cervical cancer, lung cancer and so on (Fig. S1f). Thereafter, the association between circMTCL1 and clinicopathological features was analysed by analysis of variance Chi-squared test (Table S6). As a result, the high circMTCL1 expression was significantly associated with tumour differentiation (*P* = 0.003), T stage (*P* = 0.001), lymphaticmetastasis (*P* = 0.001) and clinical stage (*P* = 0.001).

More importantly, localisation assays using RNA fluorescence in situ hybridisation (FISH) and qPCR assays revealed that circMTCL1 was mainly localised within the TU212 and LCC cell cytoplasm amplified with isolated RNA from the cytoplasmic contents and cell nuclei (Fig. 1j and k). Subsequently, a receiver operating characteristic (ROC) curve was created to assess the diagnostic cut-off value of circMTCL1 expression. Consequently, the cut-off values, the area under the curve and the sensitivity and specificity were 0.0253, 0.799 and 0.881 and 0.597, respectively (Fig. 1l). Furthermore, the log-rank test was explored to investigate whether the circMTCL1 expression level correlated with the survival of patients with LSCC. As a result, patients with higher circMTCL1 expression levels had a worse overall survival (OS) (*P* = 0.006) than those with lower circMTCL1 expression levels (Fig. 1m). Collectively, these findings demonstrated that circMTCL1 was up-regulated in LSCC cells and patients and high expression of circMTCL1 should be an independent prognositic factor for prediction of clnical outcomes in patients with LSCC.

### CircMTCL1 interacts with and inhibits C1QBP degradation via the ubiquitin–proteasome pathway

**Fig. 2:**
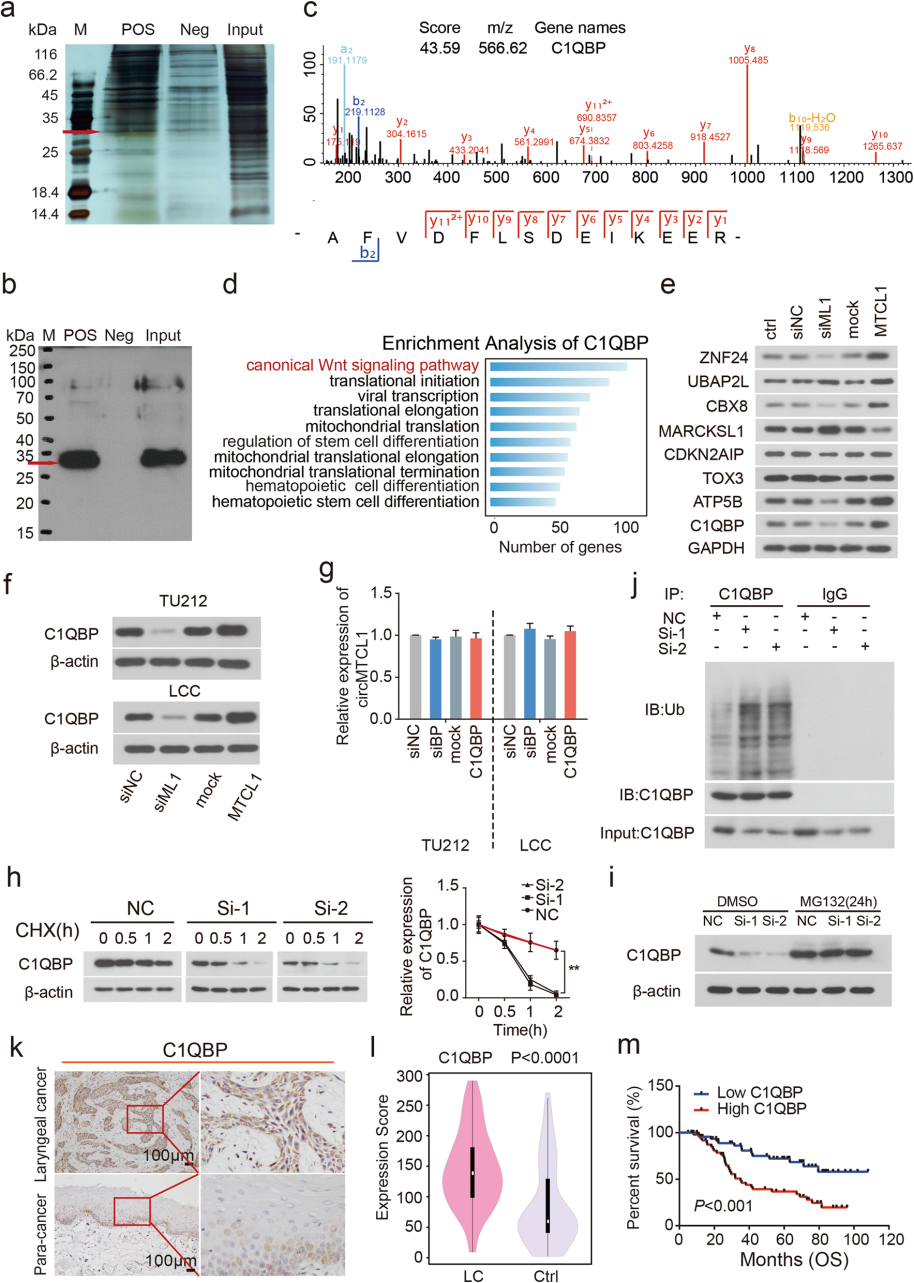
The effect of circMTCL1 on LSCC biological functions by regulating C1QBP ubiquitination. **a**, Silver staining images of PAGE gels, in which circMTCL1/proteins complexs from pull down experiments of TU212 cells were separated; the arrows represent the specific protein bands in pull down complexs by circMTCL1 sense sequence when compared with antisense sequence. **b**, Western blot assays for SDS-PAGE electrophoresis of RNA pull-down assay. **c**, The peak map of C1QBP acquired from the RNA pulldown mass spectrometry assay. **d**, KEGG analysis of the protein C1QBP, and the related signaling pathways. **e**, Western blot assays showing the effect of circMTCL1 on the proteins pulled down by target RNA probe (circMTCL1). **f**, The C1QBP expression was conducted after overexpressing or knocking down circMTCL1 by Western blot. **g**, CircMTCL1 expression was determined after overexpressing, knocking down C1QBP by qRT-PCR assays . Values are the mean ± s.d. of *n* = 3 independent experiments. **h**, Reducing the translation activity of C1QBP by silencing circMTCL1 treated by the CHX in TU212 cells (left). The quantification of C1QBP degradation rate by gray scale analysis (right). **i**, Western blot showing C1QBP protein in TU212 cells after knocking down circMTCL1 and then treated with MG132 for 24 h. **j**, Ubiquitinated C1QBP measured by immunoprecipitation with anti-C1QBP antibody or IgG control and immunoblotting with anti-ubiquitin antibody in TU212 cells after knocking down circMTCL1. **k**, IHC method was used to determine the cell localization and the expression levels of C1QBP in LSCC tissues and matched adjacent-tumor controls (*n* = 140). Scale bars = 100 μm. **l**, Violin charts illustrating the expression scores and levels of C1QBP between LSCC tissues and corresponding adjacent normal tissues (*n* = 140). Nonparametric tests and Median and 95%CI were shown. **m**, Kaplan-Meier curves displaying the impact of C1QBP on overall survival in the included LSCC cohort. *, *P* < 0.05; **, *P* < 0.01; ***, *P* < 0.001; ****, *P* < 0.0001

To further determine the regulation pattern by which circMTCL1 played a critical role in LSCC cells, RNA pull-down assays and the mass spectrometry assays were utilized to explore the potential direct binding proteins with circMTCL1. Primary, the RNA pull-down assay was performed using biotin-labelled circMTCL1 sense and antisense probes mixed with cytoplasm proteins from TU212 cells. A protein located around 32 kDa was significant enriched compared with the circMTCL1 antisense (Fig. 2a). Further mass spectrometry assays suggested that C1QBP was a potential interacting protein with circMTCL1 in LSCC cells. Thereafter, this interaction was monitored using WB analysis, and results indicated that C1QBP was found in the circMTCL1 pull-down protein complexes (Fig. 2b, c, Table S7 and Fig. S2a). Other interacting proteins of circMTCL1, such as ZNF24, UBAP2L, CBX8, MARCKSL1, CDKN2AIP, TOX3, ATP5B and C1QBP, were also investigated and found to be associated with tumorigenesis. Subsequently, the total Kyoto Encyclopaedia of Genes and Genomes (KEGG) enrichment analysis was performed for eight proteins, and a single KEGG enrichment analysis was also conducted for each protein (Fig. 2d and Fig.S2b, c, d, e, f, g, h and i). Results showed that both analyses of all eight proteins combined and the individual analysis for C1QBP revealed enrichment in the canonical Wnt signalling pathway, indicating that C1QBP is the probable binding protein of circMTCL1. Furthermore, alterations in the expression levels of eight proteins were verified using WB assays after interefering the expression of circMTCL1. Four proteins exhibited the same expression level trends, including ZNF24, CBX8, ATP5B and C1QBP, whereas MARCKSL1 demonstrated an opposite expression trend. The expression levels of three proteins remained relatively unchanged than those of the controls, including UBAP2L, CDKN2AIP and TOX3 (Fig. 2e and Fig. S3a).

To further elucidate the molecular basis of circMTCL1’s oncogenic roles, putative interactions between circMTCL1 and C1QBP were analysed. Interestingly, WB experiments demonstrated significantly alteration levels of C1QBP in TU212 and LCC cells (Fig. 2f and Fig. S3b), indicating that circMTCL1 can regulate the C1QBP expression at the post-transcriptional level. In contrast, the alteration of C1QBP expression did not affect the expression of circMTCL1, shown in Fig. 2g. Given that elevated protein levels can be caused either by increased synthesis or prolongation of its half-life, we measured the protein expression level of C1QBP without or with cycloheximide (CHX) or proteasome inhibitor MG132. The half-life of C1QBP protein was shorter in cells with down-regulated circMTCL1 expression as compared with that of controls after the CHX treatment (Fig. 2h and Fig. S3c), indicating that overexpressing circMTCL1 accelerated the C1QBP degradation in TU212 cells. Nevertheless, silencing circMTCL1 dramatically reduced the protein expression of C1QBP protein, although it remained elevated following the treatment with MG132 (Fig. 2i and Fig. S3d). Thus, circMTCL1 promotes the protein expression of C1QBP by inhibiting its degradation.

As the ubiquitin-proteasome pathway is responsible for the degradation of many proteins [26, 27], co-immunoprecipitation (co-IP) experiments were subsequently performed to measure the ubiquitination level of C1QBP when circMTCL1 was knocked down in TU212 cells. CircMTCL1 significantly inhibited ubiquitinated C1QBP in TU212 cells (Fig. 2j), suggesting that circMTCL1 inhibits C1QBP ubiquitination and up-regulates C1QBP in TU212 cells. Next, immunohistochemistry (IHC) was used to examine the expression level of C1QBP in 140 pairs of LSCC and ANM tissues (Fig. 2k). Consistent with the results from LSCC cells, higher C1QBP expression was observed in LSCC tissues compared to levels detected in the tumour-adjacent normal tissues (Fig. 2l). As expected, the prognostic analysis suggested that higher C1QBP expression was associated with shortened OS (*P* < 0.001) (Fig. 2m). Taken together, our results indicated that circMTCL1 increased the protein expression level of C1QBP by inhibiting its degradation via the suppression of the ubiquitin-proteasome pathway, however, the molecular mechanisms and underlying functions need further investigation.

### CircMTCL1 promotes LSCC cell proliferation, invasion and migration by activating Wnt/β-catenin signalling in a C1QBP-dependent manner

**Fig. 3:**
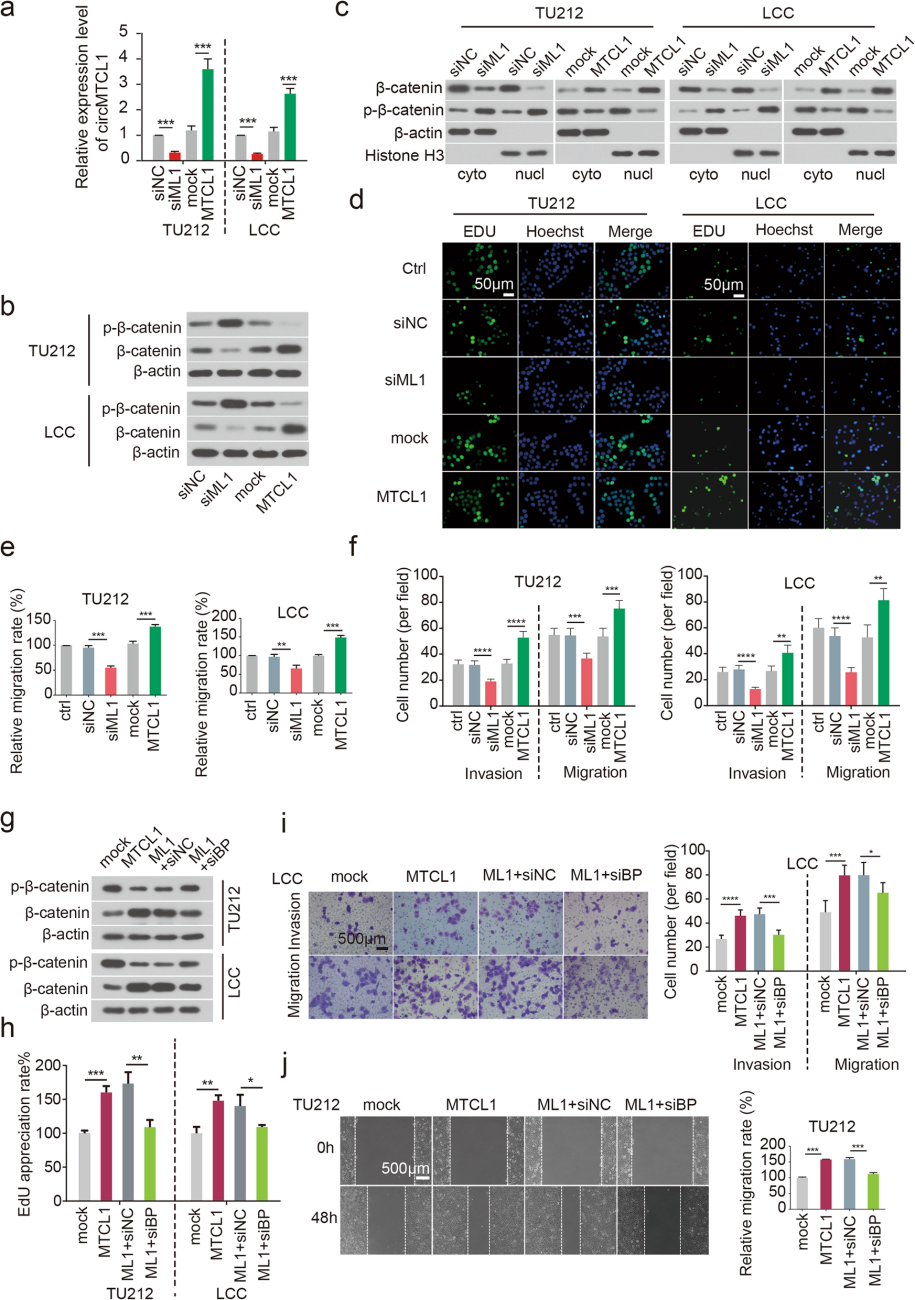
Effects of circ-MTCL1 on proliferation, invasion and migration through C1QBP in LSCC cells. **a**, The expression level of circMTCL1 was identified after overexpressing or knocking down circMTCL1 by qRT-PCR assays. Values are the mean ± s.d. of *n* = 3 independent experiments. **b**, Western blot assays were performed to evaluate the expression levels of β-catenin and p-β-catenin upon circMTCL1 silencing or overexpressing in TU212 and LCC cells. **c**, The cytoplasmic and nuclear accumulation of *β*-catenin and p-*β*-catenin were determined after ectopic or knockdown circMTCL1 by Wertern blot assays. **d**, The proliferation ability was measured upon circMTCL1 overexpressing or silencing in TU212 and LCC cells by EdU assays. Scale bars = 50 μm. **e**, Wound healing assays were performed to identify the cell motility upon circMTCL1 silencing or overexpressing in TU212 and LCC cells. **f**, The migrated cell numbers were determined after ectopic or knockdown circMTCL1 in TU212 and LCC cells. **g**,Western blot assays detected the expression of *β*-catenin and p-*β*-catenin in TU212 and LCC cells co-transfected circMTCL1 and C1QBP. **h**, CircMTCL1 rescued the proliferation ability after co-transfected circMTCL1 and C1QBP. **i**, Overexpressing circMTCL1 rescued the vertically migrated and invasive ability after silencing C1QBP. Scale bars = 500 μm. **J**, Overexpressing circMTCL1 rescued the laterally migrated ability after silencing C1QBP. Scale bars = 500 μm. *, *P* < 0.05; **, *P* < 0.01; ***, *P* < 0.001; ****, *P* < 0.0001

To identify the functional roles of circMTCL1 in LSCC, circMTCL1 was over-expressed using a Plcdh recombinant lentiviral vector or silenced circMTCL1 using small interfering RNA (siRNA) in TU212 and LCC cells (Tables S8 and S9). The qPCR analysis confirmed the transfection efficiency 48 h after transfection (Fig. 3a and Tables S10 and 11). Subsequently, combined with a previous KEGG analysis, which showed that C1QBP could be enriched in the canonical Wnt signalling pathway, these collective findings led us to presume that a novel regulatory axis involved circMTCL1 bound with C1QBP directly to regulate its activity in a concentration-dependent manner to modulate the canonical Wnt signalling pathway in LSCC. Therefore, the effects of circMTCL1 on β-catenin expression were first investigated using WB. The results demonstrated β-catenin enrichment and decreased phosphorylation when circMTCL1 was overexpressed. Consistently, circMTCL1 knockdown significantly decreased β-catenin expression and up-regulated its phosphorylation in TU212 and LCC cells (Fig. 3b and Fig. S4a). Moreover, circMTCL1 overexpression led to the cytoplasmic and nuclear accumulation of β-catenin and decreased its phosphorylation in TU212 and LCC cells, whereas circMTCL1 knockdown significantly decreased the cytoplasmic and nuclear accumulation of β-catenin and increased its phosphorylation (Fig. 3c). Thus, circMTCL1 promotes the expression of oncogenic protein β-catenin in LSCC tissues and facilitates its accumulation in cytoplasm and nucleus. To further investigate the effects of circMTCL1 on the biological characters of LSCC, EdU assays were used to determine that ectopic circMTCL1 significantly promoted cell proliferation of TU212 and LCC cells, whereas circMTCL1 silencing inhibited cell viability of these two cell lines (Fig. 3d and Fig. S4b). Furthermore, circMTCL1 overexpression significantly enhanced the number of migrated cells in both TU212 and LCC cells, whereas its knockdown repressed cell motility (Fig. 3e and Fig. S4c). The ectopic circMTCL1 expression also remarkably increased the invasiveness and motility in the LSCC cells, whereas circMTCL1 silencing had the opposite effects in TU212 and LCC cells as determined using the transwell assays (Fig. 3f and Fig. S4d). In vitro rescue experiments indicated that the effects of circMTCL1 overexpression on β-catenin expression and phosphorylation were attenuated by C1QBP knockdown as determined by WB assays (Fig. 3g and Fig. S4e). Similarly, in vitro rescue experiments also revealed that C1QBP knockdown reversed the circMTCL1 enhancing effect on LSCC biological characteristics including proliferation ability, invasion and migration ability in both TU212 and LCC cells determined by EdU (Fig. 3h and Fig. S4f), transwell (Fig. 3i and Fig. S4g) and wound healing assays (Fig. 3j and Fig. S4h). Taken togenther, circMTCL1 could promote LSCC biological functions by targeting the Wnt/β-catenin pathway in a C1QBP-dependent manner, however, the molecular mechanisms need further investigation.

### CircMTCL1 interacts with C1QBP in a direct binding manner

**Fig. 4:**
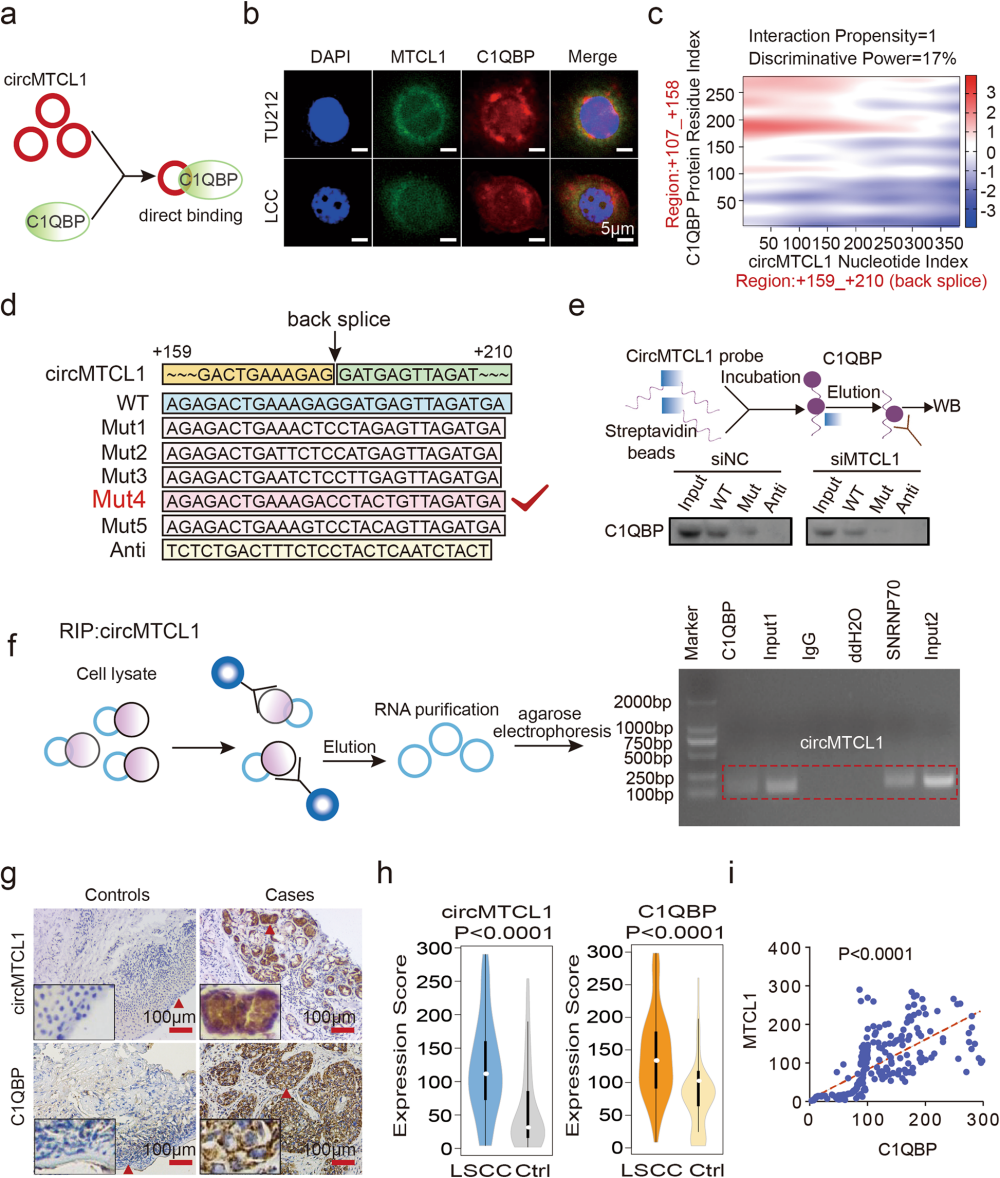
Identification of the direct interaction between circMTCL1 and C1QBP protein in LSCC cells. **a**, Schematic diagram of binding manner for circMTCL1 and C1QBP. **b**, Co-localization of circMTCL1 with C1QBP protein detected by ISH and IF assays in TU212 and LCC cells. Scale bars = 5 μm. **c**, Bioinformatics online software predicting the specific binding sequence and sites of circMTCL1 secondary structure and C1QBP protein (http://www.tartaglialab.com/). **d**, The probe information of circMTCL1 in pull-down assay. **e**, RNA pull-down followed by Western blot was conducted to examine the direct interaction of the circMTCL1-WT, circMTCL1-Mut and antisense RNA probes with C1QBP protein after circMTCL1 knockdown in TU212 cells. **f**, RIP assay was performed to verify the combination of circMTCL1 and C1QBP in TU212 cell. **g**, ISH and IHC methods were used to determine the cell localization and expression levels of circMTCL1 and C1QBP in our included clinical LSCC tissues and matched adjacent-tumor controls (*n* = 140). Scale bars = 100 μm. **h**, Violin charts displaying the expression scores of circMTCL1 and C1QBP between LSCC tissues and matched paired adjacent normal tissues (*n* = 140). Nonparametric tests and median and 95%CIs were shown. **i**, Linear correlation pattern representing a positive association of circMTCL1 expression with C1QBP expression

To further clarify the molecular mechanism of circMTCL1 interaction with C1QBP in LSCC cells, an ideograph was first used to reveal the binding manner (Fig. 4a). The co-location of circMTCL1 and C1QBP was assessed using FISH assays, revealing that circMTCL1 and C1QBP were mainly co-localised in the LSCC cell cytoplasm, supporting the possibility that circMTCL1 interacts with C1QBP (Fig. 4b and Table S12). Next, the catRAPID platform (*http://service.tartaglialab.com*) was used to examine the interaction propensity and discriminative power between the circMTCL1 nucleotide and C1QBP residue indices (Fig. 4c, Fig. S5a, b and Table S13). Due to the circMTCL1 sequence designed according to the back splicing junction, circMTCL1 (+ 159 − + 210) showed the interaction propensity (value = 1) and discriminative power (17%) for C1QBP (+ 107 − + 158). Furthermore, we synthesised the biotin-labelled circMTCL1 probe containing key binding sites with C1QBP protein (circMTCL1-WT) or a series of circMTCL1-Mut containing the corresponding mutant sequences (Fig. 4d and Table S14). Finally, the Mut4 for circMTCL1 was selected for further pull-down assay. As a result, RNA pull-down assays showed that the circMTCL1-WT probe, but not the circMTCL1-Mut probe, remarkably pulled down an endogenous cytoplasmic C1QBP protein, and this enrichment was dramatically decreased after the circMTCL1 silencing, indicating a direct binding manner between circMTCL1 and C1QBP (Fig. 4e). RNA immunoprecipitation (RIP) assays further determined that endogenous circMTCL1 co-precipitated with a C1QBP antibody and also illustrated that circMTCL1 interacted with C1QBP in a direct manner (Fig. 4f). Next, ISH and IHC were used to investigate the expression level of circMTCL1 and C1QBP in 130 pairs of LSCC and ANM tissues (Fig. 4g). As expected, higher circMTCL1 and C1QBP expressions were found in LSCC tissues as compared to ANM controls (Fig. 4h), and a positive linear correlation between circMTCL1 and C1QBP was found in this cohort (Fig. 4i). Collectively, these results demonstrated that circMTCL1 regulated C1QBP in a direct binding manner by recognising a specific sequence.

### C1QBP interacts with β-catenin and activates the Wnt/β-catenin pathway in a circMTCL1-dependent manner

**Fig. 5:**
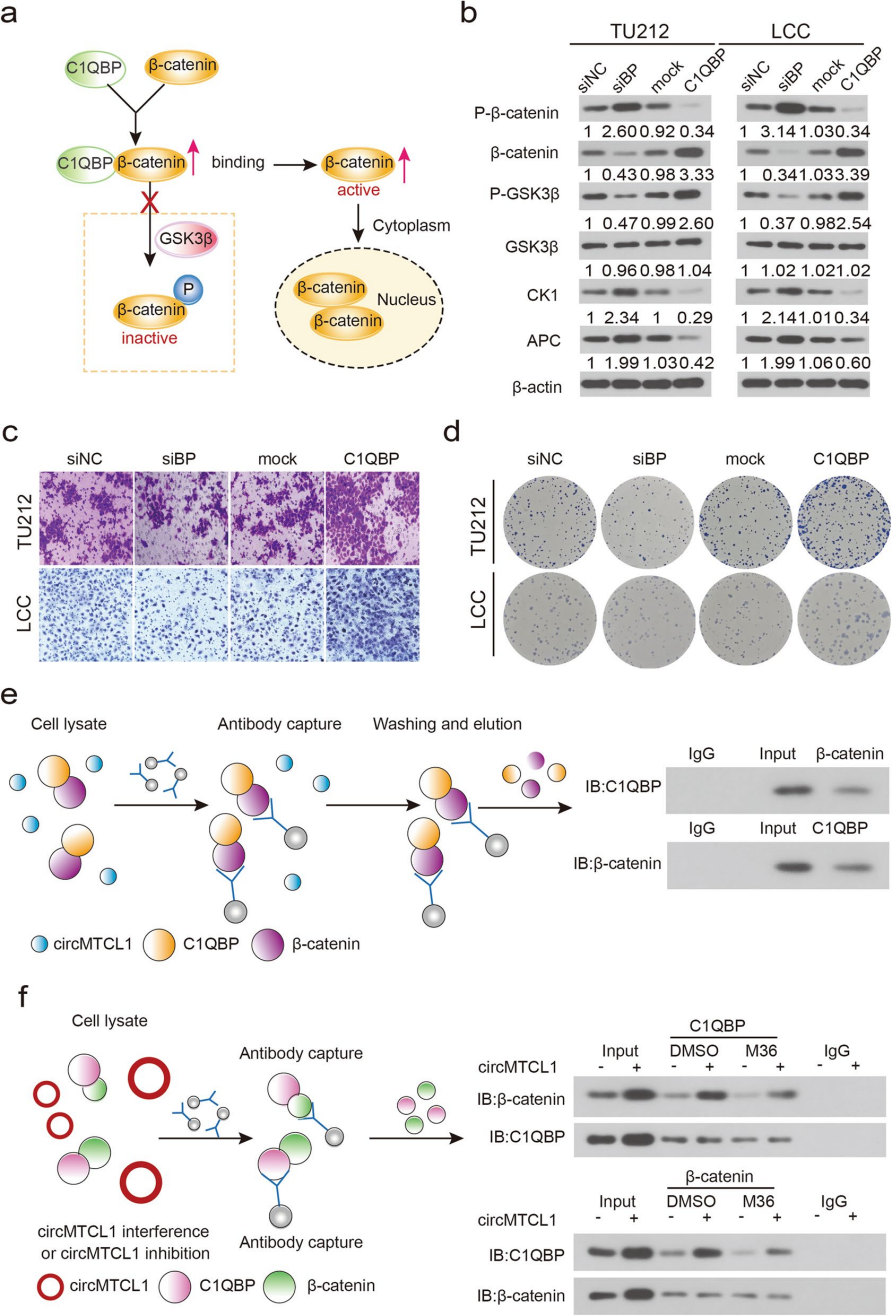
The effect of C1QBP on wnt/*β-catenin* signaling pathway regulated by circMTCL1. **a**, Schematic diagram of functioning manner between C1QBP and wnt *β-catenin* pathway regulated by circMTCL1. **b**, Western blot assays showing the expression of *β*-catenin, p-*β*-catenin, GSK3β upon C1QBP silencing or overexpressing in TU212 and LCC cell. **c**, The migrated cell numbers were determined after ectopic or knockdown C1QBP in TU212 and LCC cells. Scale bars = 500 μm. **d**, CFA assays displaying the colony-forming abilities upon C1QBP silencing or overexpressing in TU212 and LCC cells. **e**, Co-IP and western blot assays demonstrating the association of C1QBP and *β*-catenin in TU212 cells without any intervention. **f**, co-IP and western blot assays confirming the association between C1QBP and *β*-catenin after overexpressing circMTCL1 in TU212 cells. *, *P* < 0.05; **, *P* < 0.01; ***, *P* < 0.001; ****, *P* < 0.0001

Based on a previous KEGG analysis that C1QBP could be enriched in the canonical Wnt signalling pathway, we hypothesised that an interaction between circMTCL1 and C1QBP might occur. To further verify the molecular mechanism of the interaction between C1QBP and β-catenin modified by circMTCL1 and its regulation pattern, a schematic diagram was first used to illustrate this molecular mechanism (Fig. 5a). Thereafter, WB assays were used to measure the expression levels of β-catenin, phosphorylated β-catenin, glycogen synthase kinase 3 beta (GSK3β), phosphorylated GSK-3ß, *Adenomatous Polyposis Coli* (APC) and casein kinase (CK1) when circMTCL1 was overexpressed or knocked down in TU212 and LCC cells. C1QBP overexpression significantly increased the β-catenin expression, whereas its knockdown decreased its expression in TU212 and LCC cells (Fig. 5b). Consistently, ectopic C1QBP expression decreased β-catenin phosphorylation, whereas C1QBP knockdown up-regulated it (Fig. 5b). Nevertheless, no significant alteration in GSK3β was observed when C1QBP expression was altered with the GSK3β phosphorylation, exhibiting the same trend as that of C1QBP (Fig. 5b). We also observed that CK1 and APC were up-regulated when C1QBP was knocked down, whereas CK1 and APC levels decreased when C1QBP was overexpressed (Fig. 5b). Furthermore, transwell assay and clone forming assay were used to evaluate the effects of C1QBP on the biological mechanisms of LSCC. Results showed that C1QBP overexpression significantly enhanced the number of invasive and migrated cells, whereas C1QBP knockdown greatly decreased the numbers of TU212 and LCC cells (Fig. 5c, Fig. S5c). C1QBP depletion also resulted in a significant suppression in cell colony-forming abilities in both TU212 and LCC cells, and C1QBP overexpression substantially enhanced the proliferative ability of these two types of LSCC cells (Fig. 5d, Fig. S5d). Thereafter, the correlation between C1QBP and β-catenin was investigated by performing two forms of co-IP assays, and a co-IP assay without any intervention was performed to validate the endogenous association between C1QBP and β-catenin in TU212 and LCC cells (Fig. 5e). The circMTCL1 up-regulation increased endogenous interactions between C1QBP and β-catenin in LSCC cells (Fig. 5f). Collectively, these results suggested that C1QBP interacted with the oncogenic protein β-catenin and activated the Wnt/β-catenin pathway mediated by circMTCL1 in LSCC tissues.

### CircMTCL1 promotes the growth and distant metastasis of LSCC xenografts in vivo

**Fig. 6:**
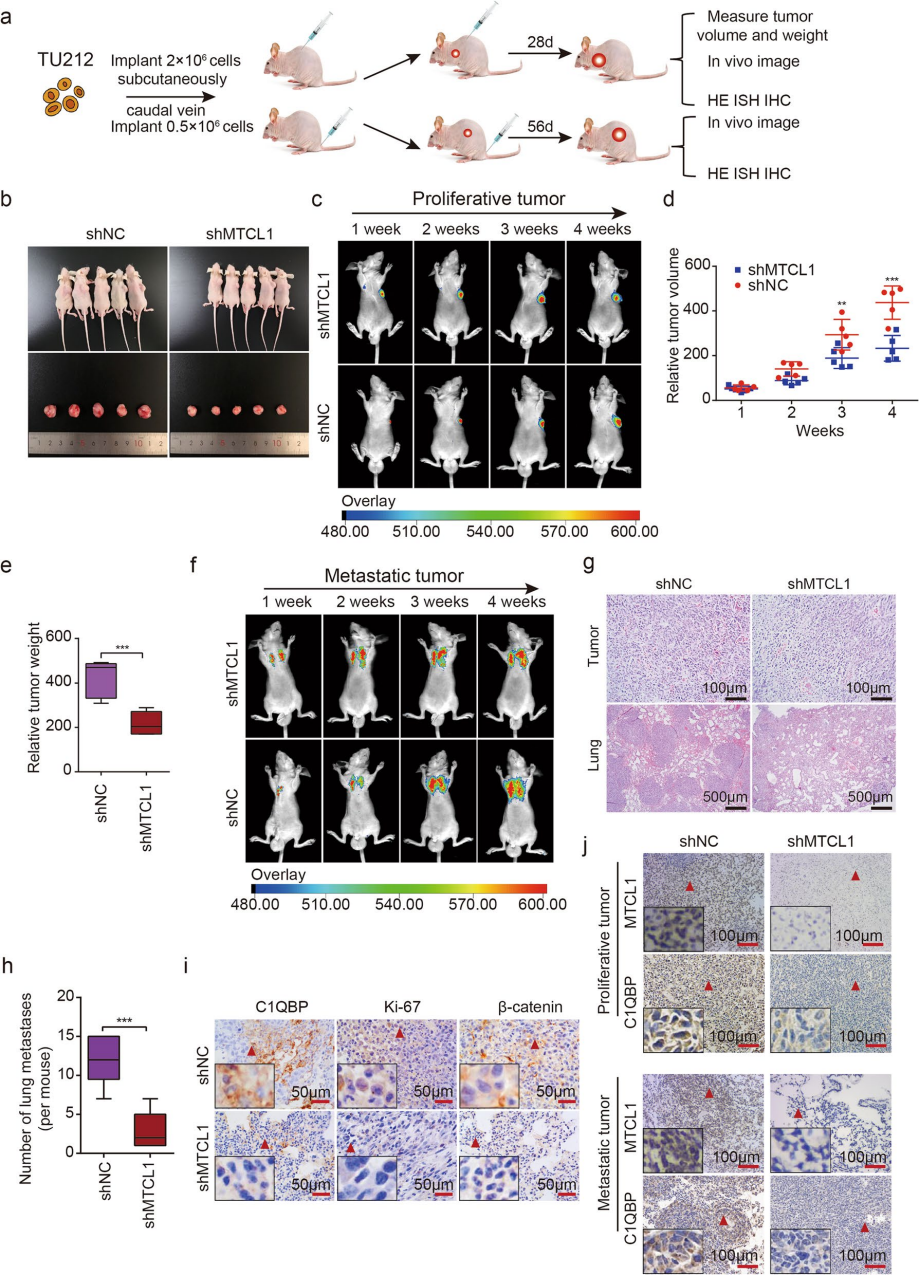
CircMTCL1 promotes growth and metastasis of LSCC xenograft in vivo. **a,** Schematic diagram of xenografts in BALB/c nude mice by inoculating TU212 cells that were stably co-transfected with sh-NC, sh-circMTCL1, respectively, at their back. Then half of the xenografts were sacrificed at the 28th day after injection and the other half were sacrificed at the 56th day after injection. **b**, Representative images of subcutaneously injecting nude mice and xenograft tumors. **c**, In vivo image of nude mice treated with subcutaneous injection of TU212 cells stably transfected with sh-circMTCL1 or sh-NC. **d**, Mean tumor volumes on different days for each group xenografts in nude mice. Data are showed as mean ± s.d, *n* = 5 for each group. **e**, Mean tumor weight of each group xenografts in nude mice. Data are showed as mean ± s.d, *n* = 5 for each group. **f**, In vivo image of xenografts formed by vein injection of TU212 cells stably transfected with sh-circMTCL1 or sh-NC. **g** and **h**, HE staining of tumors and lung metastases. Scale bars = 100 μm (upper) and 500 μm (lower). Data are showed as mean ± s.d, *n* = 5 for each group. **i**, IHC assay showing the protein expression of Ki-67 in tumors and C1QBP with *β*-catenin in lung sections of each group. Scale bars = 50 μm. **j**, ISH and IHC methods were used to identify the cell localization and expression levels of circMTCL1 and C1QBP in each group, in subcutaneous tumor and lung metastatic tumor. *n* = 5 for each group. Scale bars = 100 μm. *, *P* < 0.05; **, *P* < 0.01; ***, *P* < 0.001; ****, *P* < 0.0001

To investigate the functional role of circMTCL1 in vivo, we established xenografts in BALB/c nude mice by injecting TU212 cells stably transfected with a circMTCL1 short hairpin (shRNA) or control vector at their backs or caudal veins (Fig. 6a). The mice were sacrificed, and xenografts of tumours were removed at 4 weeks post-implantation. As expected, decreasing the circMTCL1 expression dramatically decreased LSCC cell tumorigenicity (Fig. 6b and c). Consistently, low circMTCL1expression resulted in a remarkably decreased tumour volume (Fig. 6d) and weight (Fig. 6e). Since in vitro experiments demonstrated that circMTCL1 promoted LSCC cell migration and invasion, an LSCC metastatic mouse model was constructed by injecting sh-circMTCL1 TU212 cells into the caudal vein. CircMTCL1 knockdown decreased lung metastasis (Fig. 6f). Haematoxylin and eosin (HE) staining reflected evident changes associated with circMTCL1 expression in tumours and lung tissue sections (Fig. 6g and h). Moreover, IHC assays demonstrated that circMTCL1 down-regulation evidently decreased the Ki-67 proliferative index, reduced the numbers of CD31- and CD34-positive microvessels and lowered the *β*-catenin C1QBP expression in TU212-cell-injected subcutaneous xenografts (Fig. 6i and Fig. S6a and b). IHC and ISH analyses further indicated that circMTCL1 and C1QBP were both significantly decreased in the sh-circRNA group as compared to the control group in the subcutaneous or metastatic tumours (Fig. 6j). Collectively, these results demonstrated that circMTCL1 facilitates tumour growth and metastasis in vivo.

## Discussion

**Fig. 7:**
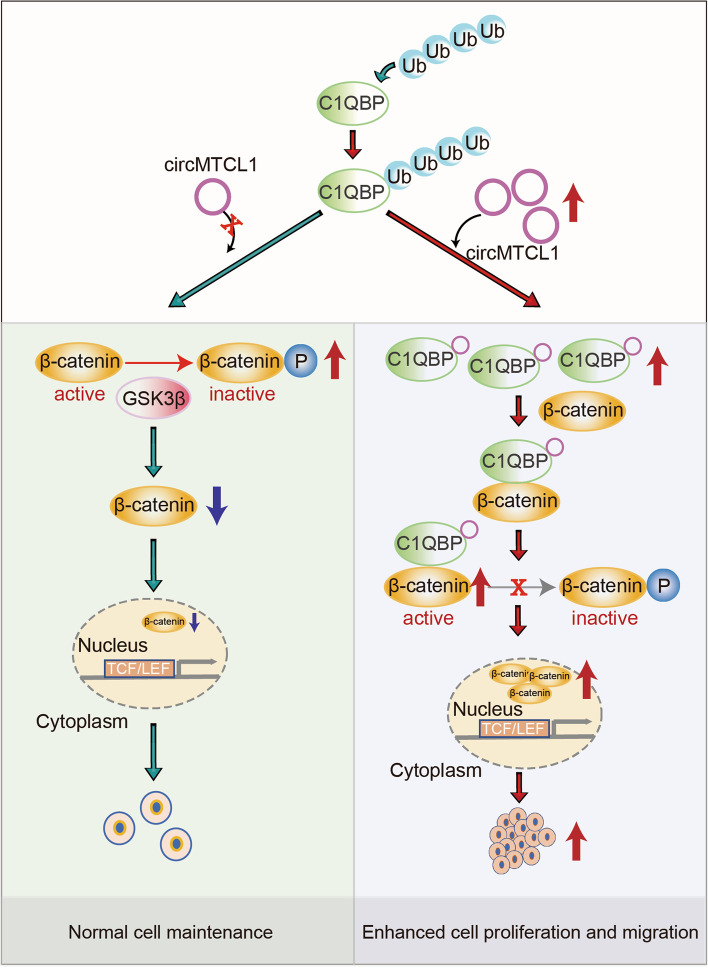
The mechanisms underlying circMTCL1 promoted LSCC progression: circMTCL1 binds to C1QBP to inhibit the degradation of C1QBP through viaubiquitin-proteasome pathway subsequently activating the wnt/β-catenin pathway

Recently, several circRNAs played crucial roles during tumorigenesis and cancer progression. However, their functional roles and underlying molecular mechanisms in LSCC remain unclear. Based on our transcriptome sequencing data, a novel molecular mechanism was identified, involving the circMTCL1-C1QBP-Wnt/*β*-catenin pathway during the occurrence and progression of LSCC (Fig. 7). CircMTCL1 interacts with C1QBP through a specific binding sequence, elevating C1QBP expression by inhibiting its degradation via the ubiquitin-proteasome pathway; this subsequently enhances the interaction between C1QBP and *β*-catenin, promoting cell proliferation, invasion and migration in vitro and in vivo. Specifically, circMTCL1 can promote interactions between C1QBP and β-catenin, preventing β-catenin phosphorylation, thereby enhancing β-catenin cytoplasmic and nuclear accumulation. Thus, our study provides new insights into how circRNAs and their corresponding RBPs regulate their target signalling pathways, which could result in novel biomarkers and targets for the LSCC diagnosis and treatment.

To date, studies involving high-throughput sequencing in LSCC were limited [28, 29], and only a few included a competitive endogenous RNA [4, 5]. However, no previous studies have investigated circRNAs that regulate LSCC progression by interacting with RBPs. In this study, through RNA pull-down assays and literature reviews, eight proteins were identified, which may be considered as the RNA binding protein of circMTCL1, including ZNF24, UBAP2L, CBX8, MARCKSL1, CDKN2AIP, TOX3, ATP5B and C1QBP [30–33]. Thereafter, through several enrichment analyses, C1QBP was found to be enriched by the canonical Wnt signalling pathway. Previous studies have mainly focused on elucidating the ways that C1QBP affects the stability and degradation of its targets; however, information on whether these changes are regulated through circular RNAs involved in tumorigenesis and development is lacking. Importantly, circMTCL1 was mainly located in the cytoplasm of LSCC cells, a finding entirely different from that of previous studies, which demonstrated that circRNAs involve in the RBP mechanism in the cell nucleus [15, 34, 35]. These results provide additional insights into the C1QBP effects on tumor progression.

In this study, we assessed the underlying mechanisms in circMTCL1’s role in regulating the C1QBP expression. A previous study showed that circ-DONSON interacts with the RBP–nucleosome remodelling factor (NURF) complex by directly combining with the SNF2L subunit in gastric cancer [36]. Furthermore, although circ-cyclin B1 (circ-CCNB1) could not directly bind to H2A histone family member X (H2AX) or p53 in breast cancer, circ-CCNB1, H2AX and p53 could form a ternary complex, which could be enhanced by circ-CCNB1 [37]. Another study reported that circ-cyclic GMP-AMP synthase (circ-cGAS) binds to the protein cGASat its double-stranded stem region (nt52–104 vs. nt149–197) [38]. In this study, the specific motif of the circMTCL1 binding site (+ 159 − + 210) was identified, with the “GAGGAT”sequence, which was consistent with the circbase database (http://www.circbase.org/). Thereafter, circMTCL1 was found to increase C1QBP expression by inhibiting its degradation through the ubiquitin-proteasome pathway. Although tremendous efforts have been made to induce crucial protein degradation in several tumours [39–41], no effective way has been established to clinically target C1QBP degradation in LSCC. Our results demonstrate that circMTCL1 directly interacts with C1QBP and inhibits its degradation, suggesting that circMTCL1 could be a potential therapeutic target to regulate the post-translational C1QBP expression in LSCC.

Furthermore, circMTCL1 was found to regulate the *β*-catenin-mediated Wnt signalling pathway through C1QBP binding. Previous studies had investigated the effects of non-coding RNAs or RNA-binding protein on the Wnt/*β*-catenin pathway. For instance, the long-coding RNA (lncRNA) nuclear-enriched abundant transcript 1 (NEAT1) was proven to enhance the *β*-catenin translocation via the RBP known as an enhancer of zeste homolog 2 (EZH2), subsequently altering negatively regulated factors of the Wnt/*β*-catenin pathway, including Axin2, ICAT, and GSK3*β* [42]. Another study showed that Hu antigen R (HuR) regulates β-catenin protein expression by stabilising its mRNA and promoting translation [43, 44]. However, the specific mechanism on how circMTCL1 or C1QBP regulated β-cateninis was inconsistent with the previous studies. In this study, GSK3*β* expression was not significantly affected by C1QBP alterations, but its phosphorylation level was, suggesting that circMTCL1 only affects phosphorylated GSK3*β*. We speculate that C1QBP binds to *β*-catenin, preventing it from being phosphorylated by GSK3*β* in the cytoplasm; subsequently, resulting in more β-catenin accumulation in the cytoplasm and nucleus, which would explain why circMTCL1 interacts with C1QBP, elevating the binding capability between C1QBP and *β*-catenin and promoting the β-catenin accumulation in the cytoplasm and nucleus.

In conclusion, our work demonstrates a novel role of circMTCL1 in regulating C1QBP expression at the post-translational level, resulting in Wnt/*β*-catenin pathway activation in LSCC. CircMTCL1 directly binds to C1QBP and subsequently elevates its expression by inhibiting its degradation, thereby promoting the accumulation of *β*-catenin in the cytoplasm and nucleus. Owing to the abundance, stability and appropriate length of the circular structure, circMTCL1 appears to be a probable biomarker for the diagnosis and a therapeutic target in LSCC.

## Supplementary Information

**Additional file 1.**

**Additional file 2.**

## Data Availability

All data generated or analyzed during this study are included in this published article and its Additional Files.

## Abbreviations

LSCC: Laryngeal squamous cell carcinoma
CircRNAs: Circular RNAs
IHC: Immunohistochemistry
ISH: In situ hybridization
ROC curve: Receiver operating characteristic curve
RIP: RNA immunoprecipitation
CircMTCL1: Microtubule cross-linking factor 1 circRNA
C1QBP: Complement C1q-binding protein
NcRNAs: Non-coding RNAs
LncRNAs: Long non-coding RNAs
MiRNAs: MicroRNAs
