# Supplementary material for: Circular RNA MTCL1 promotes advanced laryngeal squamous cell carcinoma progression by inhibiting C1QBP ubiquitin degradation and mediating beta-catenin activation
Source: Mol Cancer. 2022 Apr 2;21:92. doi: 10.1186/s12943-022-01570-4 (PMC8976408; doi:10.1186/s12943-022-01570-4)
Supplement: Supplementary file 1 — Additional file 1. [file 12943_2022_1570_MOESM1_ESM.docx]

**Additional file 1**

**This file includes:**

- **Table S1.** The top twenty circRNAs with significant fold changes both in upregulated and downregulated groups (*n* = 20)
- **Table S2.** Circular RNAs with count reads > 2
- **Table S3.** CircRNAs with the same expression trends in the cell lines as that of sequencing results
- **Table S4.** CircRNAs with the same expression trends in the sequencing tissue as that of sequencing results
- **Table S5.** Data of circRNAs cluster in Heat map analysis
- **Table S6.** The clinicopathological characteristics of 67 patients with advanced laryngeal squamous cell carcinoma (LSCC) included in our study.
- **Table S7.** The top 30 up-regulation proteins from RNA pulldown and mass spectrometry analysis
- **Table S8.** Si RNAs sequence against circMTCL1
- **Table S9.** Si RNAs sequence against C1QBP.
- **Table S10.**Primers used in quantitative real-time PCR (qRT-PCR) assays
- **Table S11.** Reaction conditions of qRT-PCR assays
- **Table S12.** Probes used in situ hybridization (ISH) assay
- **Table S13.** The binding region of circMTCL1 and C1QBP
- **Table S14.** RNA probes of RNA pull-down assays

**Table S1. The top twenty circRNAs with significant fold changes both in upregulated and downregulated groups (*n* = 20)**

| Final ID | Circbase ID | Gene | log2FC |
| --- | --- | --- | --- |
| chr17:80721840\|80739597 | hsa_circ_0000818 | TBCD | 3.31 |
| chr8:124089350\|124117704 | hsa_circ_0085439 | WDR67 | 3.29 |
| chr2:122260742\|122287901 | hsa_circ_0002374 | CLASP1 | 3.20 |
| chr17:65850055\|65850878 | hsa_circ_0045431 | BPTF | 3.16 |
| chr2:191765289\|191769893 | hsa_circ_0006662 | GLS | 2.96 |
| chr11:60899891\|60906307 | hsa_circ_0002489 | VPS37C | 1.82 |
| chr4:105439733\|105440611 | hsa_circ_0142053 | AC004053.1 | 1.81 |
| chr21:18609140\|18632376 | hsa_circ_0115690 | Circ-0115690 | 1.63 |
| chr2:61712902\|61717911 | hsa_circ_0005050 | XPO1 | 1.51 |
| chr18:8718421\|8720494 | hsa_circ_0000825 | MTCL1 | 1.48 |
| chr3:57276883\|57282379 | hsa_circ_0001317 | APPL1 | -1.69 |
| chr5:72285253\|72286691 | hsa_circ_0072954 | FCHO2 | -1.77 |
| chr16:53288349\|53308214 | hsa_circ_0000702 | CHD9 | -1.84 |
| chr14:39620949\|39628754 | hsa_circ_0031738 | TRAPPC6B | -1.86 |
| chr15:94899365\|94945248 | hsa_circ_0000660 | MCTP2 | -1.90 |
| chr12:11273608\|11276922 | hsa_circ_0140989 | TAS2R14 | -3.01 |
| chr6:13632601\|13659064 | hsa_circ_0075650 | RANBP9 | -3.07 |
| chr9:114337013\|114348445 | hsa_circ_0088030 | PTGR1 | -3.13 |
| chr2:9028147\|9098771 | hsa_circ_0008847 | MBOAT2 | -3.14 |
| chr17:39659894\|39673216 | hsa_circ_0043586 | KRT15 | -3.37 |

**Table S2. Circular RNAs with count reads > 2**

| Final ID | Circbase ID | Gene | log2 FC |
| --- | --- | --- | --- |
| chr11:60899891\|60906307 | hsa_circ_0002489 | VPS37C | 1.82 |
| chr4:105439733\|105440611 | hsa_circ_0142053 | AC004053.1 | 1.81 |
| chr21:18609140\|18632376 | hsa_circ_0115690 | Circ-0115690 | 1.63 |
| chr2:61712902\|61717911 | hsa_circ_0005050 | XPO1 | 1.51 |
| chr18:8718421\|8720494 | hsa_circ_0000825 | MTCL1 | 1.48 |
| chr3:57276883\|57282379 | hsa_circ_0001317 | APPL1 | -1.68 |
| chr5:72285253\|72286691 | hsa_circ_0072954 | FCHO2 | -1.77 |
| chr16:53288349\|53308214 | hsa_circ_0000702 | CHD9 | -1.84 |
| chr14:39620949\|39628754 | hsa_circ_0031738 | TRAPPC6B | -1.86 |

**Table S3. CircRNAs with the same expression trends in the cell lines as that of sequencing results**

| Final ID | Circbase ID | Gene | log2 FC |
| --- | --- | --- | --- |
| chr18:8718421\|8720494 | hsa_circ_0000825 | MTCL1 | 1.48 |
| chr3:57276883\|57282379 | hsa_circ_0001317 | APPL1 | -1.68 |
| chr5:72285253\|72286691 | hsa_circ_0072954 | FCHO2 | -1.77 |

**Table S4. CircRNAs with the same expression trends in the sequencing tissue as that of sequencing results**

| Final ID | Circbase ID | Gene | log2 FC |
| --- | --- | --- | --- |
| chr18:8718421\|8720494 | hsa_circ_0000825 | MTCL1 | 1.48 |
| chr3:57276883\|57282379 | hsa_circ_0001317 | APPL1 | -1.68 |

**Table S5. Data of circRNAs cluster in Heat map analysis**

| Up-regulated  circRNA | Fold Change | Down-regulated  circRNA | Fold Change |
| --- | --- | --- | --- |
| TBCD | 3.31 | APPL1 | -1.69 |
| WDR67 | 3.29 | FCHO2 | -1.77 |
| CLASP1 | 3.20 | CHD9 | -1.84 |
| BPTF | 3.16 | TRAPPC6B | -1.86 |
| GLS | 2.96 | MCTP2 | -1.90 |
| VPS37C | 1.82 | TAS2R14 | -3.01 |
| AC004053.1 | 1.81 | RANBP9 | -3.07 |
| Circ-0115690 | 1.63 | PTGR1 | -3.13 |
| XPO1 | 1.51 | MBOAT2 | -3.14 |
| MTCL1 | 1.48 | KRT15 | -3.37 |

**Table S6. The clinicopathological characteristics of 67 patients with advanced laryngeal squamous cell carcinoma included in our study.**

| **Characteristics** | **n** | **MTCL1 expression** | | ***P*** | **Adjusted** |
| --- | --- | --- | --- | --- | --- |
|  |  | **Low (%)** | **High (%)** |  | **OR (95%CI)** |
| AGE (years) |  |  |  |  |  |
| <60 | 17 | 7(21.2%) | 10(29.4%) | 0.576 | 1.0000 |
| >60 | 50 | 26(78.8%) | 24(70.6%) | 0.442 | 1.548(0.508-4.714) |
| Gender |  |  |  |  |  |
| male | 44 | 20(60.6%) | 24(70.6%) | 0.447 | 1.0000 |
| female | 23 | 13(39.4%) | 10(29.4%) | 0.391 | 1.560(0.565-4.308) |
| Location |  |  |  |  |  |
| Supraglottic | 22 | 11(33.3%) | 11(32.4%) | 0.905 | 1.0000 |
| Glottic | 24 | 11(33.3%) | 13(38.2%) | 0.778 | 0.846(0.266-2.696) |
| Subglottic | 21 | 11(33.3%) | 10(29.4%) | 0.876 | 1.100(0.332-3.640) |
| Differentiation status |  |  |  |  |  |
| Well | 14 | 12(36.4%) | 2(5.9%) | 0.003 | 1.0000 |
| poor and Moderate | 53 | 21(63.6%) | 32(94.1%) | 0.007 | 9.226(1.854-45.90) |
| T stage |  |  |  |  |  |
| T1+T2 | 20 | 16(48.5%) | 4(11.8%) | 0.001 | 1.0000 |
| T3+T4 | 47 | 17(51.5%) | 30(88.2%) | 0.002 | 8.681(2.276-33.114) |
| Lymph node metastasis |  |  |  |  |  |
| N0 | 20 | 17(51.5%) | 3(8.8%) | 0.001 | 1.0000 |
| N1 | 47 | 16(48.5%) | 31(91.2%) | 0.001 | 10.0000 |
| Clinical stage |  |  |  |  | 10.703(2.654-43.166) |
| III | 24 | 19(57.6%) | 5(14.7%) | 0.001 | 1.0000 |
| IV | 43 | 14(42.4%) | 29(85.3%) | 0.001 | 7.787(2.316-26.180) |

T, tumor; N, lymphatic node.

**Table S7. The top 30 upregulation proteins from** **RNA pulldown and mass spectrometry analysis.**

| No | Protein name | Gene | Fold change | Regulation |
| --- | --- | --- | --- | --- |
| 1 | Cleavage stimulation factor subunit 2 | CSTF2 | 119.4 | up |
| 2 | Ribosomal protein 63, mitochondrial | MRPL57 | 104.3 | up |
| 3 | Thyroid hormone receptor-associated  protein 3 | THRAP3 | 99.4 | up |
| 4 | Adenosine kinase | ADK | 89.6 | up |
| 5 | Protein virilizer homolog | KIAA1429 | 84.2 | up |
| 6 | Myosin light chain 3 | MYL3 | 82.0 | up |
| 7 | Matrin-3 | MATR3 | 81.4 | up |
| 8 | Dolichyl-diphosphooligosaccharide-  protein glycosyltransferase subunit 1 | RPN1 | 77.8 | up |
| 9 | TOX high mobility group box family  member 3 | TOX3 | 73.3 | up |
| 10 | ATP synthase subunit d, mitochondrial | ATP5H | 73.3 | up |
| 11 | Interleukin enhancer-binding factor 3 | ILF3 | 72.7 | up |
| 12 | Complement component 1 Q  subcomponent-binding protein | C1QBP | 70.3 | up |
| 13 | TATA-binding protein-associated  factor 2N | TAF15 | 68.7 | up |
| 14 | Ubiquitin-associated protein 2-like | UBAP2L | 68.0 | up |
| 15 | Protein FAM207A | FAM207A | 65.8 | up |
| 16 | Zinc finger protein 24 | ZNF24 | 65.1 | up |
| 17 | CDKN2A-interacting protein | CDKN2AIP | 64.6 | up |
| 18 | CDKN2AIP N-terminal-like protein | CDKN2AIPNL | 63.0 | up |
| 19 | MARCKS-related protein | MARCKSL1 | 62.8 | up |
| 20 | Chromobox protein homolog 8 | CBX8 | 62.7 | up |
| 21 | LINE-1 retrotransposable element  ORF1 protein | L1RE1 | 59.8 | up |
| 22 | 60S ribosomal protein L26-like 1 | RPL26L1 | 58.9 | up |
| 23 | ATP synthase subunit beta | ATP5B | 54.5 | up |
| 24 | Protein IWS1 homolog | IWS1 | 58.3 | up |
| 25 | Protein kinase C delta-binding protein | PRKCDBP | 58.1 | up |
| 26 | Fatty acid-binding protein, epidermal | FABP5 | 57.8 | up |
| 27 | Phostensin | PPP1R18 | 57.6 | up |
| 28 | DNA topoisomerase 1 | TOP1 | 56.9 | up |
| 29 | Protocadherin Fat 3 | FAT3 | 56.7 | up |
| 30 | 28S ribosomal protein S14,  mitochondrial | MRPS14 | 55.5 | up |

**Table S8. Si RNAs sequence against circMTCL1**

| **Si and NC** | **Sequence (5’-3’)** |
| --- | --- |
| Circ-MTCL1(si-1) | AAAGAGGATGAGTTAGATGAA |
| Circ-MTCL1(si-2) | GACUGAAAGAGGAUGAGUUTT |
| Circ-MTCL1(si-3) | AGACUGAAAGAGGAUGAGUUA |
| NC | UUCUCCGAACGUGUCACGUTT  ACGUGACACGUUCGGAGAATT |

**Table S9. Si RNAs sequence against C1QBP.**

| Si and NC | Sequence (5’-3’) |
| --- | --- |
| C1QBP-homo-777  C1QBP-homo-469  C1QBP-homo-648 | CCUUAUAUGACCACCUAAUTT  GUCACUUUCAACAUUAACATT  GGAUGAGGUUGGACAAGAATT |
| NC | UUCUCCGAACGUGUCACGUTT  ACGUGACACGUUCGGAGAATT |

| Primer | Sequence (5’-3’) |
| --- | --- |
| CircMTCL1-Forward | ACAGGCCCTCCAGAATGAGC |
| CircMTCL1-Reverse | TTCTCGCCGAAGTTCCTGCA |
| C1QBP-Forward | GTTGGACAAGAAGACGAGGCTGAG |
| C1QBP- Reverse | CCTTCCATTCAGACTCGCCAGTG |
| VPS37C-Forward | CCATGAGGATGCTGTCCCAC |
| VPS37C- Reverse | ATCGCCTCCGAGTCATTCTG |
| AC004053.1-Forward | CACCTGACCTCTCATCAACAGACT |
| AC004053.1-Reverse | TGCGAGTTGTCTCTGCTGCT |
| Circ-0115690-Forward | ACACCTGCCCAGCAGAATTA |
| Circ-0115690-Reverse | AGCTGGTTAACTTACATTTGCCTT |
| XPO1-Forward | AATTGTCAACAAGTTAGGGGGA |
| XPO1-Reverse | TGCACCAATCATGTACCCCA |
| APPL1-Forward | CCCTTATATGTGCCTGACCCA |
| APPL1-Reverse | CGGCTATATCTATTAATCGCAGC |
| FCHO2-Forward | AACTCAGGCCCTCCAGAAATC |
| FCHO2-Reverse | GTCATTGACCTGGAGTATGCC |
| CHD9-Forward | TGCTTCGTGTGAGAATGCTGT |
| CHD9-Reverse | CTGACCAATTCTGTGGCAACG |
| TRAPPC6B-Forward | TGTGGCTTAATCAGAGGTGGC |
| TRAPPC6B-Reverse | CTTGTCCCACTCGAAACCCC |
| GAPDH-Forward | GGACCTGACCTGCCGTCTAG |
| GAPDH-Reverse | TAGCCCAGGATGCCCTTGAG |

**Table S10. Primers used in quantitative real-time PCR (qRT-PCR) assays**

**Table S11. Reaction conditions of qRT-PCR assays**

| circRNA and mRNA | |
| --- | --- |
| **Relative reagents** | **1×system (20 μL)** |
| 10×RT Buffer | 2 μL |
| Enzyme Mix | 0.8 μL |
| Primer Mix | 2 μL |
| Mutiscribe TM Reverse Transcriptase  RNA+DEPC H_2_O | 1 μL  14,2 μL |
| Program: 25℃/10min 37℃/120min 85℃/5min Maintain at 4 ℃ | |
| **qPCR** | |
| **Relative reagents** | **1×system (20 μL)** |
| SYBR | 10 μL |
| DYEⅡ | 0.4 μL |
| Forward Primer (10μΜ) | 0.8 μL |
| Reverse Primer (10μΜ) | 0.8 μL |
| cDNA  DEPC H_2_O | 2μL  6μL |
| Program: 50℃/2min 95℃/5min [95℃/5 sec 60℃/34sec 95℃/15sec]×40cycles Maintain at 4℃ | |

**Table S12. Probes used in situ hybridization (ISH) assay**

| FITC-labed probe | Sequence (5’-3’) |
| --- | --- |
| CircMTCL1 | GTTCATCTAACTCATCCTCTTTCAGTCTCTCC |

**Table S13. The binding region of circMTCL1 and C1QBP**

| No. | Protein region | RNA region |
| --- | --- | --- |
| 1 | 57-108 | 26-77 |
| 2 | 57-108 | 109-160 |
| 3 | 57-108 | 126-177 |
| 4 | 107-158 | 159-210 |
| 5 | 57-108 | 84-135 |
| 6 | 57-108 | 226-277 |
| 7 | 57-108 | 101-152 |
| 8 | 57-108 | 284-335 |
| 9 | 32-83 | 159-210 |
| 10 | 151-202 | 159-210 |
| 11 | 182-233 | 159-210 |
| 12 | 57-108 | 209-260 |
| 13 | 76-127 | 159-210 |
| 14 | 57-108 | 34-85 |
| 15 | 101-152 | 159-210 |
| 16 | 57-108 | 276-327 |
| 17 | 82-133 | 159-210 |
| 18 | 157-208 | 159-210 |
| 19 | 57-108 | 76-127 |
| 20 | 132-183 | 159-210 |

**Table S14. RNA probes of RNA pull-down assays**

| Probe | Sequence (5’-3’) | Modified method |
| --- | --- | --- |
| CircMTCL1-WT | AGAGACTGAAAGAGGATGAGTTAGATGA | 5’ Biotin |
| CircMTCL1-Mut | AGAGACTGAAAGACCTACTGTTAGATGA | 5’ Biotin |
| CircMTCL1-Antisense | TCTCTGACTTTCTCCTACTCAATCTACT | 5’ Biotin |
