# Supplementary material for: Circular RNA MTCL1 promotes advanced laryngeal squamous cell carcinoma progression by inhibiting C1QBP ubiquitin degradation and mediating beta-catenin activation
Source: Mol Cancer. 2022 Apr 2;21:92. doi: 10.1186/s12943-022-01570-4 (PMC8976408; doi:10.1186/s12943-022-01570-4)
Supplement: Supplementary file 2 — Additional file 2. [file 12943_2022_1570_MOESM2_ESM.docx]

**Additional file2**

**This file includes:**

- **Fig S1.** The expression trends of circRNAs in the sequence data, LSCC cell lines and other cancers
- **Fig S2.** The proteins from RNA pulldown mass spectrometry assay
- **Fig S3.** The expression level of proteins from RNA pulldown mass spectrometry assay
- **Fig S4.** The effect of circMTCL1 on LSCC cell proliferation, invasion and migration in a C1QBP dependant manner
- **Fig S5.** The association between circMTCL1 and C1QBP
- **Fig S6.** The impact of circMTCL1 on growth and metastasis of LSCC xenograft in vivo

**
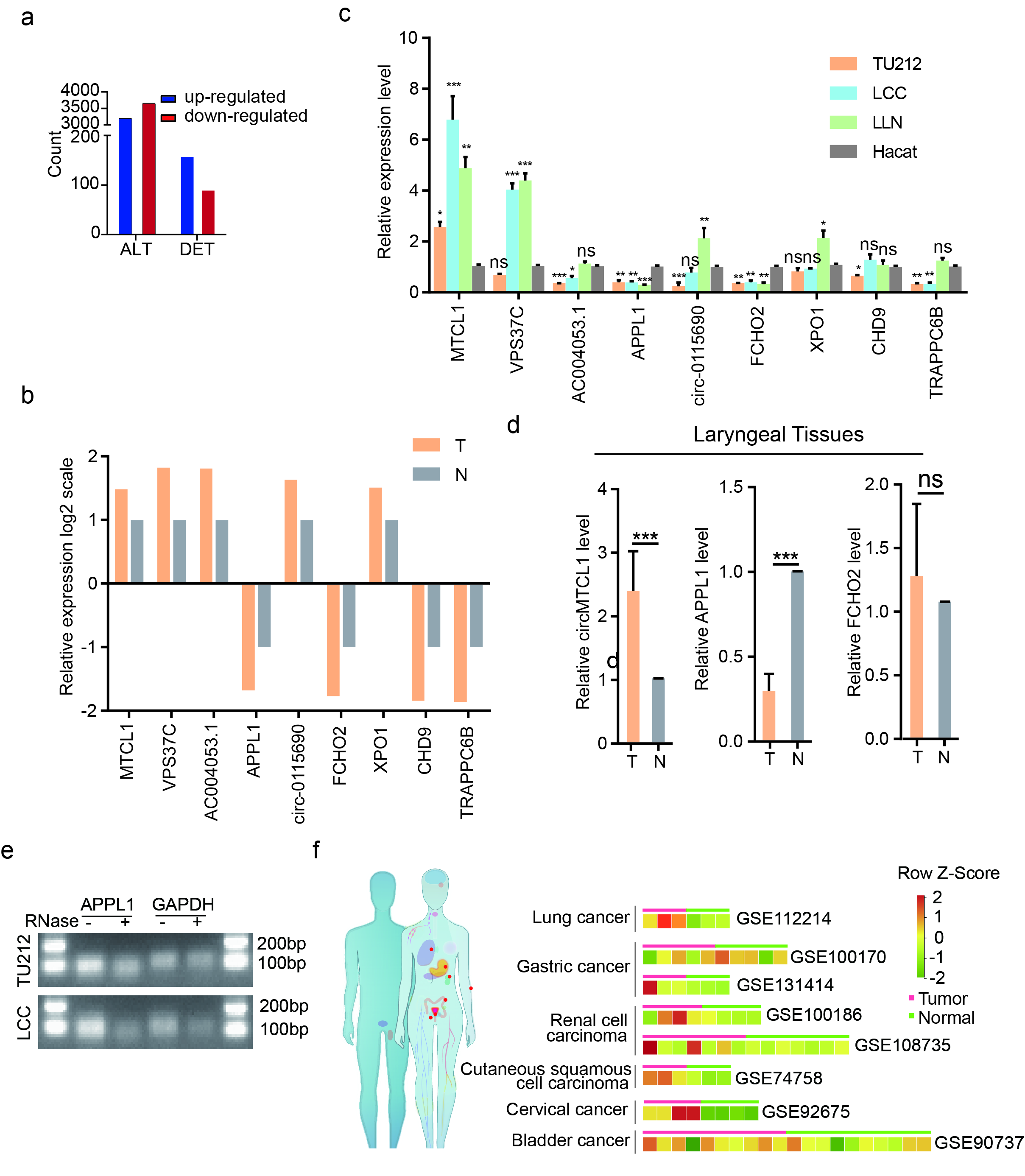
**

**Fig. S1 The expression trends of circRNAs in the sequence data and LSCC cell lines**

**a**, The histogram showing all detected(ALT) and differentially expressed (DET) circRNAs of the sequencing data. **b**, The expression trends of genes with count reads>2 in the sequencing results. **c**, qRT-PCR assays showing the expression level of the included genes in normal control HaCaT cell and LSCC cells including TU212, LCC, LLN. Values are the mean ± s.d. of *n* = 3 independent experiments. **d**, Verification of circRNAs expression trends in the laryngeal cancer tissues using qRT-PCR assays. Values are the mean ± s.d. of *n* = 3 independent experiments. **e**, RNase R digestion showing the tolerance of APPL1 to digestion. **f**, GEO website (<https://portal.gdc.cancer.gov/>) showing the expression level of circMTCL1 in the other cancers. *, *P* < 0.05; **, *P* < 0.01; ***, *P* < 0.001

**
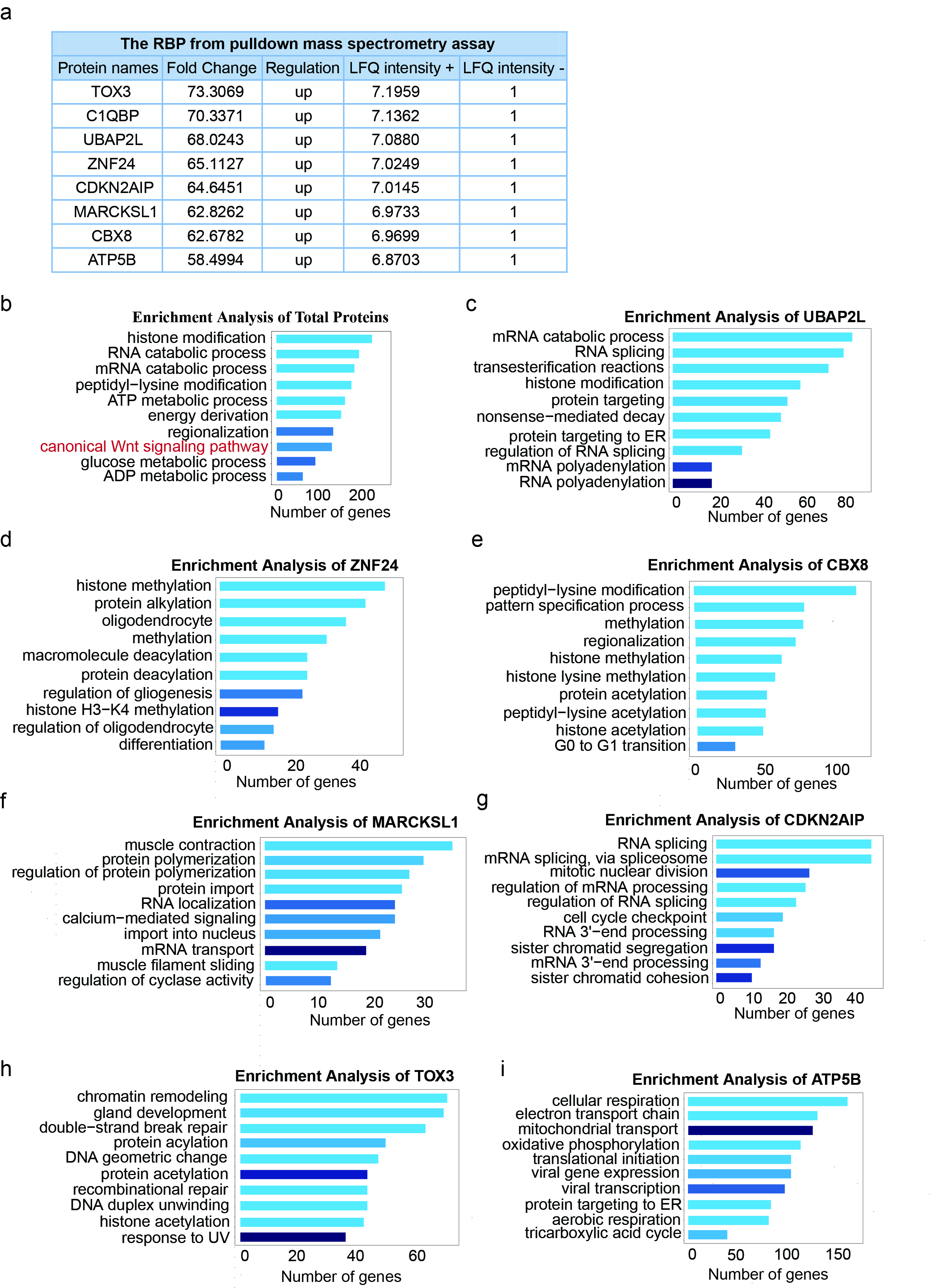
**

**Fig. S2** **The proteins from RNA pulldown mass spectrometry assay**

**a**, The table showing the selected proteins from the pull down Mass spectrometry assay. LFQ represents label-free quantitative. **b**, KEGG analysis of all the proteins from RNA pulldown mass spectrometry assay. **c-i**, KEGG analysis of single protein from RNA pulldown mass spectrometry assay respectively.


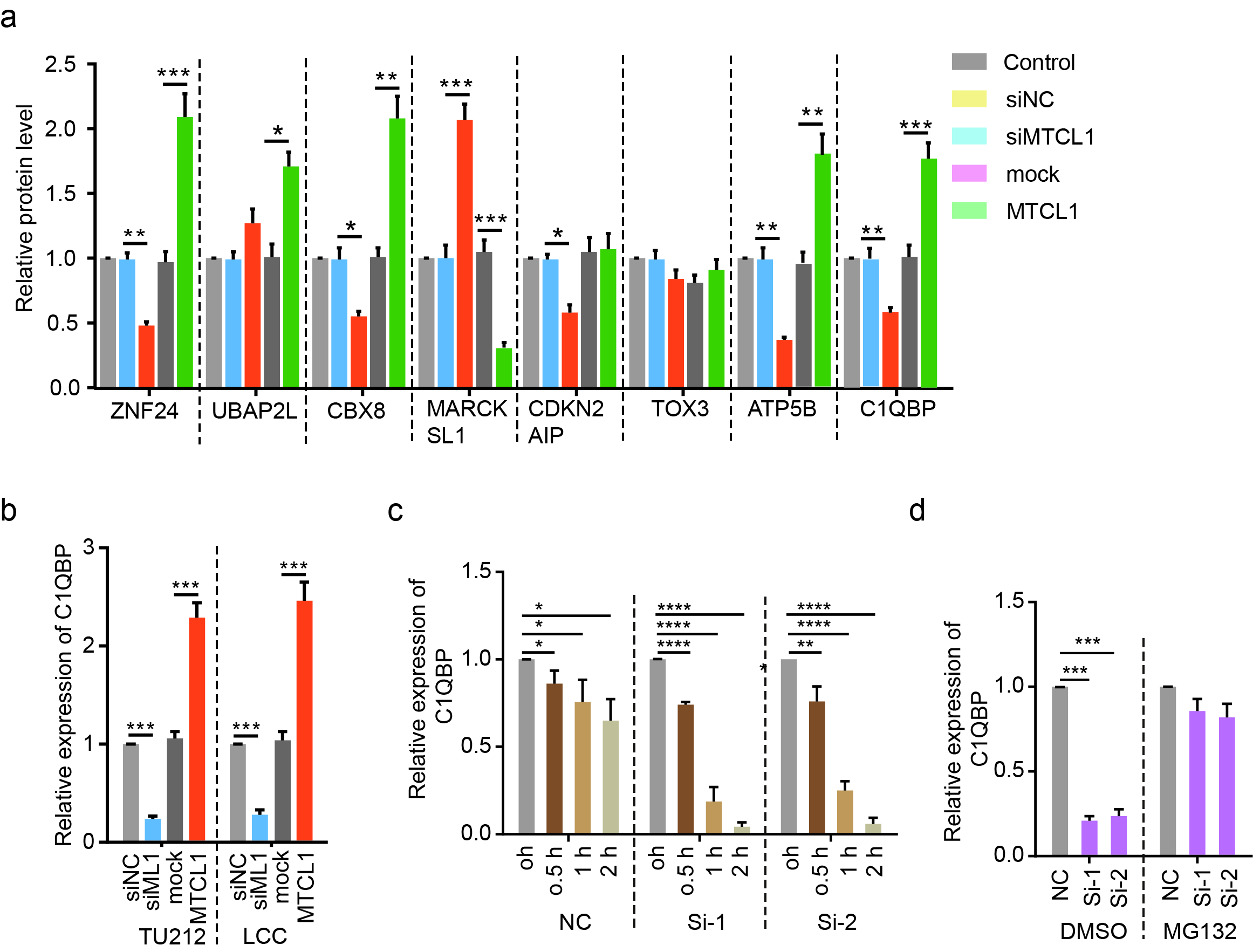


**Fig. S3 The expression levels of proteins from RNA pulldown mass spectrometry assay**

**a**, Western blot assays showing the effect of circMTCL1 on the proteins pulled down by target RNA probe. Values are the mean ± s.d. of *n* = 3 independent experiments. **b**, Western blot assay was performed to evaluate the expression level of C1QBP after overexpressing, knocking down circMTCL1. Values are the mean ± s.d. of *n* = 3 independent experiments. **c**, Western blot assays showing C1QBP expression after ectopic or knockdown circMTCL1 in TU212 cells and then treated with CHX for the indicated time. Values are the mean ± s.d. of *n* = 3 independent experiments. **d**, The expression level of C1QBP was determined after overexpressing or knocking down circMTCL1 by western blot assays and then treated with MG132 for 24h. Values are the mean ± s.d. of *n* = 3 independent experiments. *, *P* < 0.05; **, *P* < 0.01; ***, *P* < 0.001; ****, *P* < 0.0001


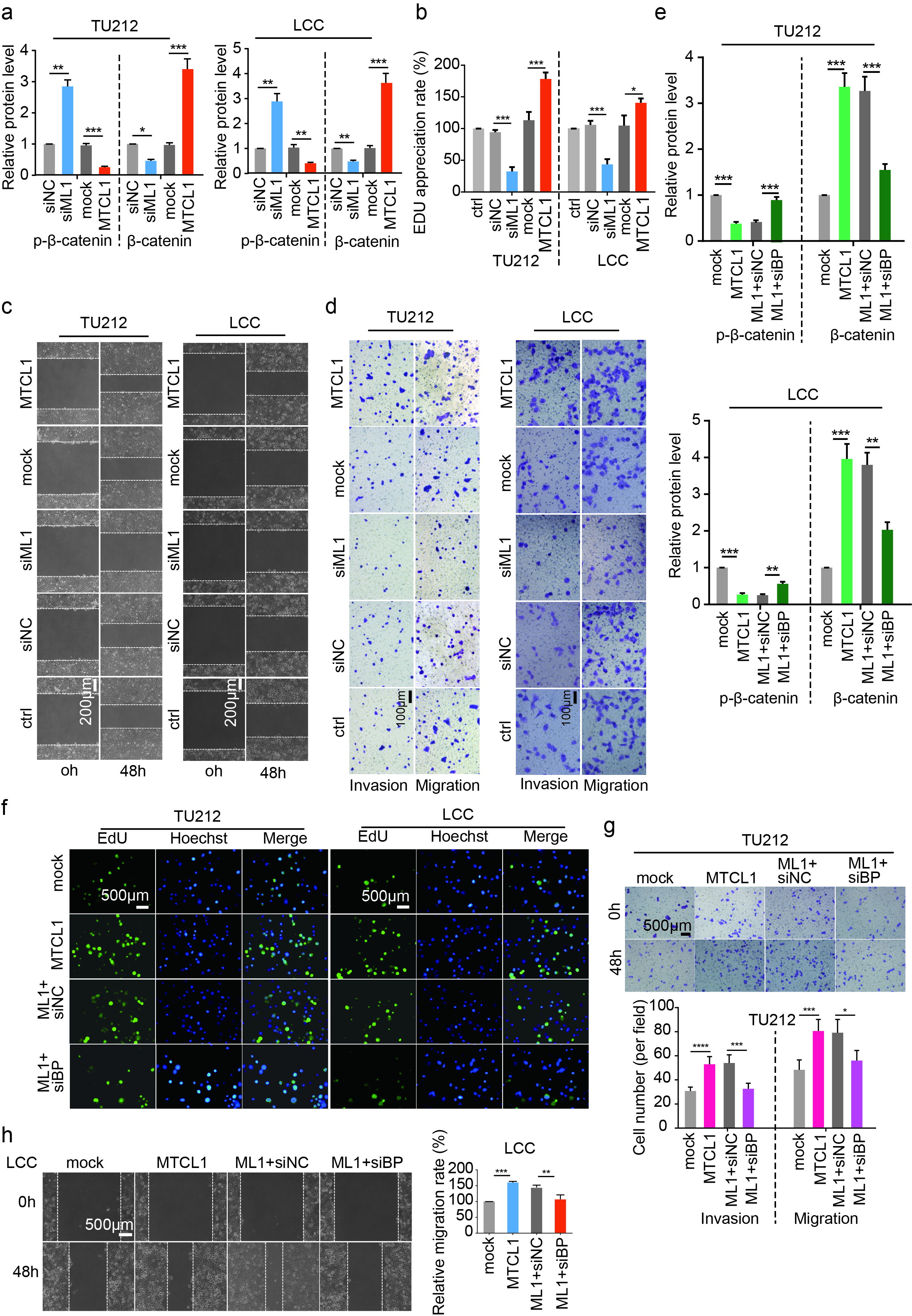


**Fig. S4** **The effect of circMTCL1 on LSCC cell proliferation, invasion and migration in a C1QBP dependant manner**

**a**, Western blot assays were performed to evaluate the expression levels of β-catenin and p-β-catenin after overexpressing or knocking down circMTCL1 in TU212 cells and LCC cells. Values are the mean ± s.d. of *n* = 3 independent experiments. **b**, The histogram of EDU assays showing the proliferation ability upon circMTCL1 silencing or overexpressing in TU212 and LCC cells. **c**, Wound healing assay was performed upon circMTCL1 overexpressing or silencing in TU212 and LCC cells. Scale bars = 200μm. **d**, The migrated cell numbers were determined after ectopic or knockdown circMTCL1 in TU212 and LCC cells. Scale bars = 100μm. **e**, Western blot assays detected the expression of *β*-catenin and p-*β*-catenin in TU212 and LCC cells co-transfected circMTCL1 and C1QBP. Values are the mean ± s.d. of *n* = 3 independent experiments. **f**, CircMTCL1 rescued the proliferation ability after co-transfected circMTCL1 and C1QBP. Scale bars = 500μm. **g**, Overexpressing circMTCL1 rescued the vertically migrated and invasive ability after silencing C1QBP. Scale bars = 500μm. **h**, Overexpressing circMTCL1 rescued the laterally migrated ability after silencing C1QBP. Scale bars = 500μm. *, *P* < 0.05; **, *P* < 0.01; ***, *P* < 0.001; ****, *P* < 0.0001


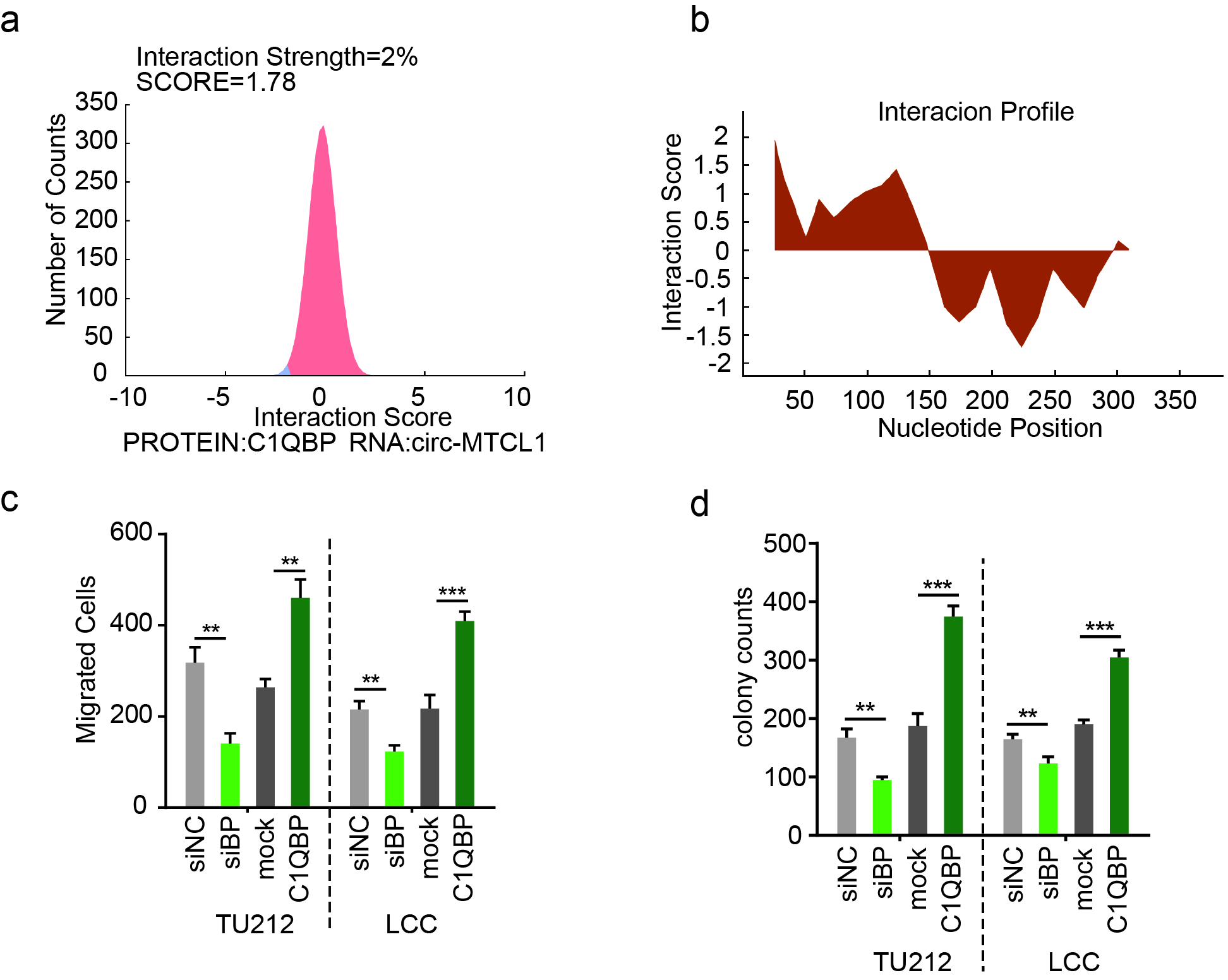


**Fig. S5 The association between circMTCL1 and C1QBP**

**a** and **b**, catRAPID website (<http://service.tartaglialab.com>) evaluating the interaction strength, scores and profile between circMTCL1 and C1QBP. **c**, Wound healing assays were performed to identify the cell motility upon C1QBP silencing or overexpressing in TU212 and LCC cells. **d**, CFA assays displaying the colony-forming abilities upon C1QBP silencing or overexpressing in TU212 and LCC cells. *, *P* < 0.05; **, *P* < 0.01; ***, *P* < 0.001


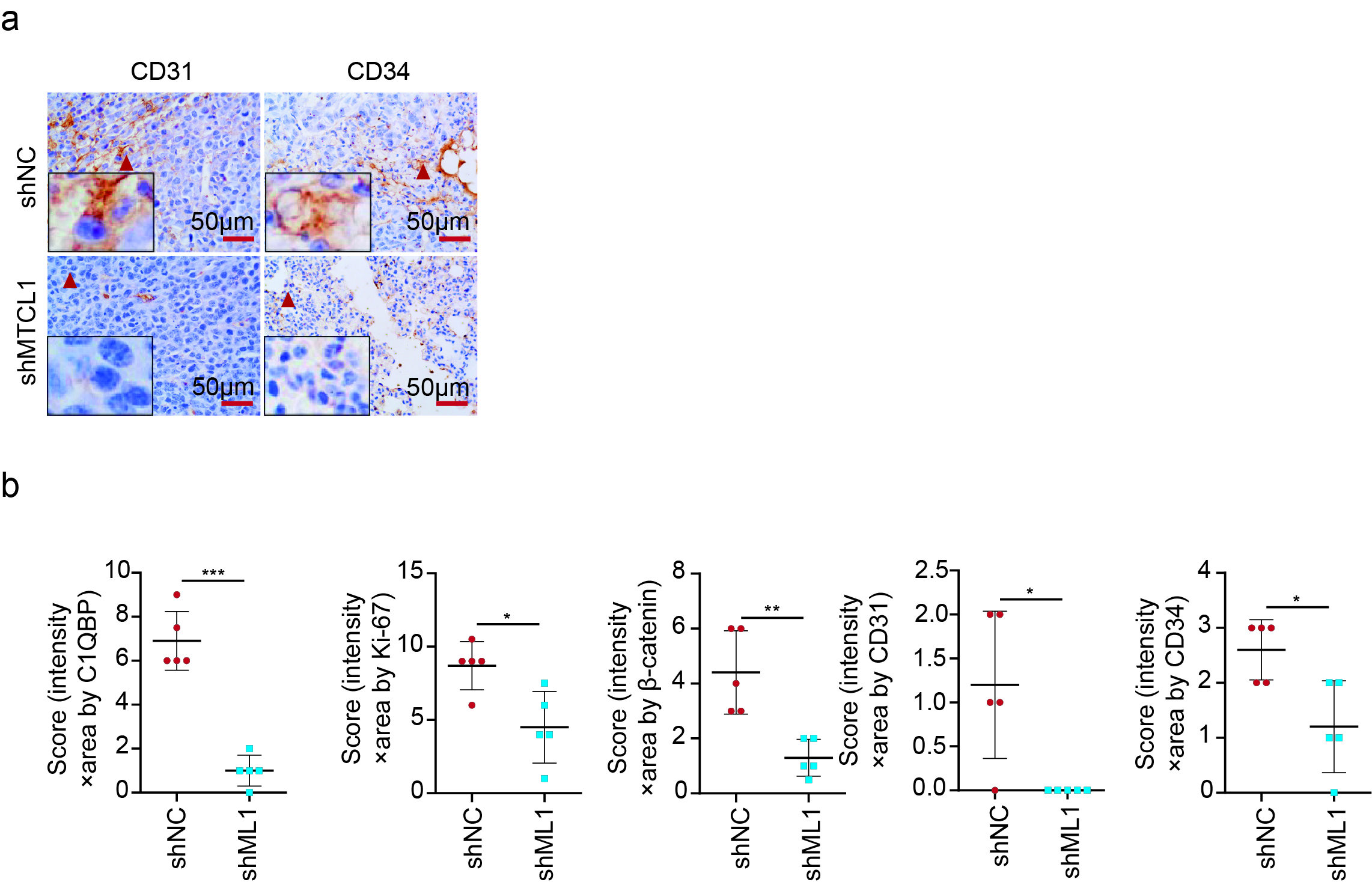


**Fig. S6 The impact of circMTCL1 on growth and metastasis of LSCC xenograft in vivo**

a and b, IHC method was used to determine the cell localization and expression levels of CD31 in tumors and CD34 in lung sections after knocking down circMTCL1. Scale bars = 50μm. Data are showed as mean ± s.d, *n* = 5 for each group. *, *P* < 0.05; **, *P* < 0.01; ***, *P* < 0.001
